# BMP-2-Driven Osteogenesis: A Comparative Analysis of Porcine BMSCs and ASCs and the Role of TGF-β and FGF Signaling

**DOI:** 10.3390/biology14060610

**Published:** 2025-05-26

**Authors:** Roman Taday, Pascal Jungbluth, Sebastian Zensen, Thomas Krakau, Joachim Windolf, Michèle J. Hoffmann, Vera Grotheer

**Affiliations:** 1 Department of Orthopedic and Trauma Surgery, University Hospital Düsseldorf, Moorenstraße 5, 40255 Düsseldorf, Germany; roman.taday@med.uni-duesseldorf.de (R.T.); sebastian.zensen@uk-essen.de (S.Z.); thomas.krakau@med.uni-duesseldorf.de (T.K.); joachim.windolf@med.uni-duesseldorf.de (J.W.); 2 Department of Urology, Medical Faculty and University Hospital Düsseldorf, Heinrich Heine University Düsseldorf, 40255 Düsseldorf, Germany; michele.hoffmann@hhu.de; 3 Department of Orthopedics, Medical School, Klinikum Bielefeld—Mitte, University Medical Center OWL, Bielefeld University, 33604 Bielefeld, Germany; vera.grotheer@uni-bielefeld.de

**Keywords:** osteogenic differentiation potential, mesenchymal stromal cells, BMP-2, adipose derived stromal cells, bone-marrow-derived stromal cells, MAPK, pp38, TGF-β, FGF

## Abstract

Mesenchymal stromal cells (MSCs), such as bone marrow-derived cells (BMSCs) and adipose-derived cells (ASCs), are key candidates for bone regeneration therapies but have not yet been integrated into standard clinical practice due to heterogeneity in their osteogenic capacities. This study investigated the osteogenic differentiation of porcine BMSCs and ASCs by analyzing BMP-2-induced receptor expression and the effects of inhibiting BMP, TGF-β, and FGF signaling pathways. While pBMSCs underwent osteogenesis in standard differentiation medium, pASCs required BMP-2 stimulation to initiate this process. BMP signaling inhibition via dorsomorphin suppressed osteogenic differentiation, but this effect was reversed by co-inhibition of TGF-β or FGF signaling. Notably, simultaneous inhibition of TGF-β and FGF in the presence of BMP-2 optimized osteogenic differentiation in both pMSC types. In pASCs, successful differentiation correlated with early activation of p38 MAPK and Wnt signaling pathways, with BMP-2 serving as a primary driver, while TGF-β and FGF pathways acted as modulators. These findings highlight the importance of signaling context and MSC tissue origin in bone formation and suggest that tailored modulation of BMP, TGF-β, and FGF signaling will be necessary in future in vivo applications to maximize the regenerative potential of MSC-based therapies.

## Simple Summary

Large bone defects caused by trauma or disease are difficult to heal and currently treated using bone grafts, which have several drawbacks like limited availability and risk of complications. Stem cells, especially those from bone marrow (BMSCs) or fat tissue (ASCs), offer promising alternatives for bone regeneration. However, the success of such therapies varies greatly depending on cell type, preparation, and interaction with the body. In this study, we examined how stem cells from pigs—an established model for human bone healing—respond to different biological signals during bone formation. We found that fat-derived stem cells (pASCs) need an additional protein, BMP-2, to begin bone development, whereas bone marrow cells (pBMSCs) can start the process more effectively on their own. Our results suggest that BMP-2 works by activating specific cellular pathways (p38 and Wnt) needed for bone growth. Interestingly, blocking other signals (TGF-β and FGF) further boosted bone development when combined with BMP-2. These findings highlight the complex but crucial role of cellular signaling in bone healing and suggest how we might improve stem cell therapies for critical bone defects. Better understanding these mechanisms may lead to safer, more effective treatments for patients in the future.

## 1. Introduction

Critical-size bone defects, such as those that can occur after severe trauma or a bone tumor, represent a particular challenge in orthopedics and trauma surgery [1,2,3]. Current clinical practice considers cancellous bone autografts as the gold standard for large bone defects. Yet, this method still faces critical disadvantages, such as a limited donor supply, prolonged donor site morbidity, hemorrhage, and limited tissue yield and quality [2,4,5,6]. Alternatives, like allogenic bone grafts, are likewise afflicted with significant risks of causing a host immune response, the transmission of infectious diseases, and possible side effects on the mechanical and biological properties of the graft [4,7,8]. Meanwhile, a proven therapeutic alternative for bone repair and regeneration is displayed by multipotent mesenchymal stromal cells (MSCs) composited with an appropriate scaffold [6,9,10]. MSCs can be isolated from numerous tissue types, whereby BMSCs (bone marrow-derived stromal cells) and ASCs (adipose-derived stromal cells) have been proven to be particularly suitable MSC sources for bone tissue engineering [6,10,11,12,13,14,15,16].

But despite these successes, the therapeutic application of human ASCs or BMSCs has not yet become a standardized clinical reality, since the published studies are characterized by great variability. The osteogenic regeneration potential of MSCs depends on various parameters, including the tissue origin, applied cell count and preparation, senescence, and interaction with bone graft substitutes [11,17,18,19]. To guarantee the most effective, individualized, and safe clinical application of the optimal MSC type to heal critical-size bone defects in humans, it is crucial to understand the molecular mechanisms of the osteogenic differentiation of MSCs in sufficient detail. Therefore, systematically conducted MSC applications in preclinical models are urgently needed, for which pigs have been proven to be the most suitable animal model [12,20]. Although the pig is an excellent model for studying MSC-based bone regeneration, significant differences between the species necessitate thorough understanding, allowing researchers to better tailor regenerative treatments for improved efficacy and safety in clinical applications through comprehensive comparison of molecular mechanisms. In contrast to human ASCs (hASCs), the osteogenic differentiation of pASCs is dependent on the addition of BMP-2 (bone morphogenetic protein 2) [21].

This raises the question of why pASCs are dependent on BMP-2, and which signaling pathways and respective receptor expressions are essential for their sufficient osteogenic differentiation. Osteogenic differentiation relies on precise coordination of multiple signaling cascades, particularly TGF-β and BMP pathways, which are crucial for MSC osteogenesis and are dysregulated in bone disorders. BMP-2 has been used clinically for treating bone healing disorders [15,22,23,24]. FGF signaling is vital in early osteogenesis and maintaining balance between osteoblasts and osteoclasts [25,26,27,28], interacting with Wnt, MAPK, and BMP pathways, leading to stimulating effects on osteoprogenitors and inhibitory effects on terminal osteoblastic differentiation. The p38 MAPK pathway, part of noncanonical TGF-β signaling, is essential for osteoblast maturation [24,29], while early Wnt activation drives MSC differentiation into osteoblast progenitors and BMP promotes the maturation of osteoblasts and ossification of the extracellular matrix [30,31]. The key molecules that have to be induced or inhibited to optimize the differentiation potential and to achieve comparable differentiation levels for various MSC types still remain uncertain [21]. The study aims to assess the cell type-specific osteogenic differentiation potential of BMP-2 induced pASCs and pBMSCs by investigating the influence of BMP, TGF-β, and FGF signaling on osteogenesis through selective inhibition of these pathways. We analyzed the effect of selective inhibition of BMP, TGF-β, and FGF signaling on the osteogenic differentiation of pBMSC and pASC. The goal was to gain insights into the significance of these signaling cascades for bone formation, ultimately improving the adaptation and differentiation capacity of transplanted cells in bone tissue engineering strategies.

## 2. Materials and Methods

### 2.1. Porcine ASC and BMSC Harvest

Primary ASCs were obtained from the subcutaneous fat tissue and bone marrow of Göttingen minipigs and the subcutaneous fat tissue of domestic pigs (Danish sow, Piètrain boar). Some bone marrow and adipose tissues were taken from the experimental animals after euthanasia, which were part of another study in our laboratory (file number 84-02.04.2013.A459 of LANUV NRW), and some were from slaughtered animals whose fat tissue was obtained from a local abattoir. A skin sample with subcutaneous fat tissue of about 20 g was excised from the dorsal upper leg of the pig. The bone marrow was obtained by puncturing the iliac crest under aseptic conditions [7]. Since tissue pieces from already euthanized or slaughtered animals were used for the experiments, no animal experiment permit was necessary (§ 7 para. 2, p. 3 TierSchG, Germany). The animal experiment reference number is G 459/13,2013. The pigs were 6 to 30 months old and weighed about 25 to 35 kg (Table S4).

### 2.2. Methods of MSC Preparation

#### 2.2.1. Isolation of pACS

The obtained tissue pieces were kept refrigerated in PBS (phosphate-buffered saline) with the addition of penicillin (200 U/mL), streptomycin (0.2 mg/mL), and amphotericin B (2.5 μg/mL) until further direct processing. The connective tissue and blood vessels of fat lobules were removed under sterile conditions to a size of less than approx. 0.5 cm^3^ each. To cleave the collagen and dissolve the cell compounds, the fat tissue was incubated for 45 min with sterile filtered collagenase solution (0.2% collagen type I, 1.5% BSA (bovine serum albumin)) at a ratio of 1:2 in a shaking bath preheated to 37 °C. The digested fatty tissue was filtered (Teflon or nylon sieve (100–250 μm)), and the cell suspension was centrifuged at 300× *g* for 10 min. After the removal of the upper solid and lower liquid phases, the remaining cell pellet was resuspended in PBS and centrifuged again. The supernatant was aspirated, and the cell pellet was resuspended with penicillin (200 U/mL), streptomycin (0.2 mg/mL), and amphotericin B (2.5 μg/mL) and supplemented with proliferation medium containing DMEM (Dulbecco’s Modified Eagle Medium; gibco^®^ ref 41965-039, lot 1924339) (+4.5 g/L of D-Glucose, +L-Glutamine, and L-Pyruvate) and 10% FBS (fetal bovine serum) (PAN^TM^ Biotech Sera Plus). This cell suspension was placed in a T75 cell culture flask, which was cultivated at 37 °C and 5% CO_2_ in a humidified incubator. The medium was changed after 24 and 48 h. All cell lines or donors were treated independently.

#### 2.2.2. Isolation of pBMCs

To prevent coagulation, the obtained bone marrow aspirate was mixed with heparin at a 1:3 ratio. The BMSCs were isolated using density gradient centrifugation at 840× *g* for 20 min, as the different cell populations differed in their density. Bone marrow aspirate was added to 5 mL of Percoll and 3 mL of 0.9% NaCl solution, creating a density gradient for separation. Density gradient centrifugation separated the cell suspension into four phases of decreasing density: plasma, mononuclear cell phase, Percoll, and a cell pellet of erythrocytes and granulocytes. The BMSCs were located within the mononuclear cell phase. The cell fraction was washed with PBS^2−^ and centrifuged at 300× *g* for 5 min. The supernatant was then discarded, and the cell pellet was resuspended with a cell culture medium and transferred to a T75 cell culture flask. As with the ASCs, cultivation was carried out in a humidified atmosphere at 37 °C and 5% CO_2_. After 24 h and 48 h of cell isolation, the medium was changed after regular light microscopic monitoring. All cell lines or donors were treated independently.

### 2.3. Phenotypical Analysis of pMSCs Using Flow Cytometry

To characterize the antigen phenotype of the pMSCs, they were examined using flow cytometry (FACS, fluorescence-activated cell sorting). The BMP-2-induced pASC-specific surface antigen receptor expression was analyzed every second day (on days 0, 1, 3, 5, 7, 9, 13, 17, 19, 21, 23, 25, and 28). The respective conjugated antibodies were used for the expressions of ALK3 (Cat. No.: AF436), ALK 5 (Cat. No.: FAB5871), ALK6 (Cat. No.: FAB5051A), TGF-β2-RII (Cat. No.: FAB532P), ALK7 (Cat. No.: FAB77491A), ALK2 (Cat. No.: AF637), ALK4 (Cat. No.: MAB2221), and BMPR-II (Cat. No.: AF811) (by R&D Systems, Minneapolis, MN, USA), and the pASCs and pBMSCs were compared for their expressions of the specific surface antigens CD45 (Cat. No.: MCA1568GA, Bio-Rad, Hercules, CA, USA), HLA-DR (human leukocyte antigen–antigen D-related surface molecule) (Cat. No.: MCA2314F, Bio-Rad, Hercules, CA, USA), CD29 (Cat. No.: 561,496, BD Pharmingen, Franklin Lakes, NJ, USA), CD79alpha (Bio-Rad, Cat. No.: MCA2538GA), CD14 (Cat. No.: MCA1568GA, Bio-Rad, Hercules, CA, USA), CD31 (Cat. No.: AF3387, R&D Systems, Minneapolis, MN, USA), CD105 (Cat. No.: NB110-58718APC, Novus Biologicals, Minneapolis, MN, USA), CD26 (, Cat. No.: NB600-552APC, Novus Biologicals, Minneapolis, MN, USA), CD73 (, Cat. No.: AF4488, R&D Systems, Minneapolis, MN, USA), CD90 (Cat. No.: 559,869, BD Pharmingen, Franklin Lakes, NJ, USA), CD34 (Cat. No.: 81289, abcam, Cambridge, UK), and CD44 (Cat. No.: 5531, BD Pharmingen, Franklin Lakes, NJ, USA). According to the manufacturer’s specifications, the appropriate staining concentrations were used [32]. The expressions of the surface antigens were analyzed using the flow cytometer from BD FACS Scan and evaluated with BD CellQuest™ (BD Biosciences, Franklin Lakes, NJ, USA).

### 2.4. Seeding and Osteogenic Differentiation of pASCs and pBMSCs

Seeding was carried out in 6- or 24-well plates for osteogenic differentiation. For the osteogenic differentiation medium (OM), DMEM (+4.5 g/L D-Glucose, +L-Glutamine, and L-Pyruvate), FBS 10%, penicillin (100 U/mL), streptomycin (0.1 mg/mL), dexamethasone (100 nmol), L-Ascorbin-2-Phosphate (50 µmol), and β-Glycerophosphate (10 mM) [33] were used. The cells destined for the BMP-2 test group were treated with osteogenic differentiation medium (OM), with the addition of 450 ng/mL BMP-2 (PeproTech, Hamburg, Germany), based on a previous titration study [34]. In the subsequent experiment series, the pASCs and pBMSCs were treated with specific inhibitors that prevent the signal transduction of the corresponding pathway to investigate the importance of FGF, BMP, and TGF-β signaling. The pASCs and pBMSCs were always osteogenically differentiated in passage 3 with OM +/− BMP-2 and with inhibition of a signaling pathway for up to 28 days. The optimal inhibitor concentrations were evaluated by adding the appropriate inhibitor in ascending concentrations, as previously described (Figures S1–S3) [35]. BGJ398 was used as an inhibitor of FGFR 1–3 at a concentration of 0.5 μM. Dorsomorphin was supplemented at a concentration of 0.5 μM to inhibit the effects of BMP-initiated signaling pathways by the inhibition of BMPR-I. The TGF-β signaling pathway was inhibited by co-incubation with 1 μM SB431542, which is an inhibitor of ALK5, ALK4, and ALK7 [36]. The osteogenic differentiation medium (OM) was changed twice a week.

### 2.5. Analysis of Osteogenic Differentiation Detection Using Alizarin Red S Staining and Quantification Using Cetylpyridinium Chloride

Alizarin red S is a dye that selectively binds to calcium deposits in the extracellular matrix [37]. Alizarin red S staining was performed on days 0, 14, 21, and 28. The adherent cell monolayers cultured in 6-well plates were washed with 1 mL PBS (phosphate-buffered saline) and fixed with 4% paraformaldehyde for 15 min, rinsed 2 times with PBS, incubated for 20 min at 37 °C with alizarin red S (0.5% in aqua dest., pH 4.1), and washed with distilled H_2_O until the supernatant was colorless. The stained monolayers coated with PBS were visualized by microscopy (Zeiss Axiovert 200 microscope). This process was followed by a quantitative destaining procedure using 10% (*w*/*v*) cetylpyridinium chloride in 10 mM sodium phosphate, pH 7.0, for 25 min at room temperature [32,38]. The optical density of the supernatant with redissolved alizarin red S dye was measured at **λ** = 600 nm against 10% cetylpyridinium chloride solution as a zero-value adjustment [35,38]. The proportion of calcium deposited into the extracellular matrix was thus indirectly quantified photometrically. A sixfold determination was carried out.

### 2.6. Western Blot Analysis of Protein Expression in pACS

Over a period of 28 days, Western blot analysis was performed on the key protein expressions and receptors of BMP (Smad4; Cat. No. GTX112980, GeneTex Inc., Irvine, CA, USA), FGF (FGFR2; Cat. No. PA5-14651, ThermoScientific, Waltham, MS, USA), MAPK (pp38; Phospho-p38 MAPKinase (Thr180/Tyr182) AK, Cat. No. 9211, Cell Signaling Technology^®^, Danvers, MS, USA), and Wnt (β-Catenin; Cat. No. 16051, abcam^®^, Cambridge, UK) signaling. The cells were seeded in 6-well plates. To determine the protein concentrations, a BCA protein assay (Pierce^TM^ BCA Protein Assay Kit; Thermo Scientific, Prod. No 23225, Lot No. SA244529) was used. The absolute protein concentration was measured photometrically in relation to a defined standard by the protein-mediated reduction of Cu^2+^ to Cu^+^ in an alkaline environment (biuret reaction). The absorbance of the samples was measured in the Victor^TM^3 (Perkin Elmer 1420 Multilabel Counter) at **λ** = 562 nm and set in relation to the standard to calculate the absolute protein concentration. The sodium dodecyl-polyacrylamide gel electrophoresis (SDS-PAGE) method with Laemmli buffer (4 × Tris-glycine SDS sample buffer, 252 mmol Tris–HCl, pH 6.8, 40% glycerin; 8% SDS, 0.01% bromophenol blue + 20% mercaptoethanol) was used. After the proteins in the gel were separated according to their size, the proteins were transferred to a PVDF membrane for further analysis. The Bio-Rad Trans-Blot^®^ Turbo^TM^ (Bio-Rad ChemiDoc^TM^ MP Imaging System) was used (25 V, 2.5 A, 15 min). Primary antibody diluted in a blocking buffer was added to the membrane overnight at 4 °C and, afterward, washed with TBS-T (Tris-buffered saline with Tween). The specific anti-Rabbit second antibody (Dako Denmark, Glostrup, Denmark), diluted in TBS-T at a ratio of 1:1000, was then incubated together with the anti-protein marker (1:4000; 0.025% anti-Western marker) for 1 h at room temperature on the Taumel-roller mixer (Fröbel Labortechnik RM5-V 1750). After washing again with TBS-T, the chemiluminescence reaction of the antibody–protein complexes was recorded using the ChemiDoc Imaging System from Bio-Rad and subsequently quantified in Image Lab 6.0., and GAPDH (glyceraldehyde-3-phosphate dehydrogenase) (Cat. No. NBP2-27103, Novus Biologicals^®^, Minneapolis, MN, USA) was used as a reference gene. For the Western blot analyses, we harvested proteins on days 0, 7, 14, 21, and 28 during the differentiation process, both with and without BMP-2. Cell culture samples were analyzed randomly and not pooled on these days, and the results were combined. Due to variations in differentiation rates among donors, we combined the results from days 7 and 14, as well as those from days 21 and 28, categorizing a mid and late phase.

### 2.7. Statistical Analysis

The statistical analysis and graphical representation of the results were carried out using Graphpad Prism^®^ 5.01. software (Dotmatics, Bishop’s Stortford, UK). An experimental design and replicate overview is provided in supplemental data (Table S5). For the analysis of optical density measurements of Alizarin Red staining, receptor expression, and protein expression, a two-way ANOVA followed by Bonferroni’s post hoc test was used for statistical evaluation. The data were arithmetically averaged and compared using a one-factorial analysis of variance with Bonferroni correction. The arithmetic mean, mean, and standard deviation were determined using the Row statistic of the integrated test procedure. The significance level was set at *p* ≤ 0.05.

## 3. Results

### 3.1. Characterization of pASCs and pBMSCs

**Figure 1:**
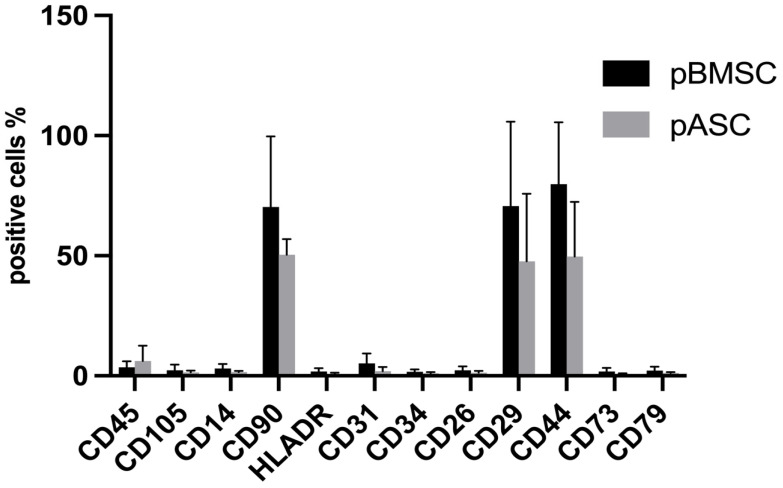
Comparison of antigen expression. pASC and pBMSC were analyzed in passage 3 by flow cytometry for the expression of the surface antigens. Positive cells % refers to the percentage of pMSCs detected by flow cytometry that expressed the surface antigen. pASCs: *n* = 12, pBMSCs: *n* = 4.

Porcine MSCs from adipose tissue and bone marrow were immunophenotypically examined for their antigen expressions of certain clusters of differentiation (CD) using flow cytometry [36,37]. Overall, the pASCs and pBMSCs showed similar expression patterns of the investigated surface antigens (Figure 1, Figures S4 and S5). The pASCs and pBMSCs were positive for the surface markers CD29, CD90, and CD44 with expressions of more than 70% (>50% for pBMSCs). For CD14, CD26, CD31, CD34, CD45, CD73, CD79, and HLA-DR, the expression levels were, on average, below 5% for all MSCs examined.

### 3.2. Comparison of Osteogenic Differentiation of pASCs and pBMSCs with Addition of BMP-2

#### 3.2.1. Osteogenic Differentiation of pASCs with or Without BMP-2

**Figure 2:**
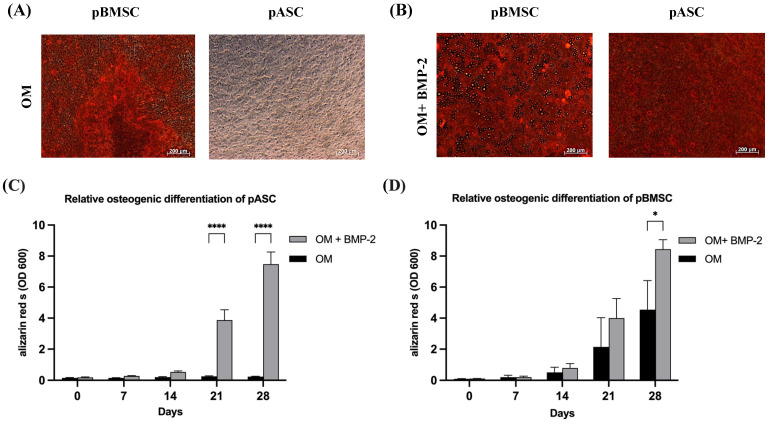
Osteogenic differentiation of pASCs and pBMSC. (**A**) Exemplary representation of light microscopic detection of alizarin red S staining after 28 days of differentiation with osteogenic differentiation medium (OM) in pBMSC and pASC. (**B**) Alizarin red S staining after 28 days with osteogenic differentiation medium supplemented with BMP-2 (450 ng/mL). Scale bar = 200 um. (**C**,**D**) The alizarin red S dye was quantified photometrically (OD = optical density). pASC and pBMSC were cultured with osteogenic differentiation medium (OM) with or without (+/−) BMP-2 for up to 28 days. The alizarin red S staining was quantified photometrically. Significant differences between the treatment groups are marked **** *p* ≤ 0.001, * *p* ≤ 0.05; pASC (*n* = 18), pBMSC (*n* = 4), OD = optical density, BMP-2 (450 ng/mL).

From day 21, osteogenic differentiation was significantly elevated in the pASCs treated with BMP-2 compared to the pASCs without BMP-2. Alizarin red S staining was used to measure the potential of osteoblasts to deposit calcium in the extracellular matrix [37]. In pASCs only incubated in OM, no significant osteogenic differentiation was detectable (Figure 2). Conclusively, the matrix mineralization in the group without the addition of BMP-2 remained at an average of 0.245 on day 21 and 0.217 on day 28, while in the pASCs with the addition of BMP-2, it was significantly superior, averaging 3.289 on day 21 and 6.545 on day 28. The two test groups, therefore, differed significantly from day 21 onward (*p* < 0.001) (Figure 2, Table S1).

#### 3.2.2. Osteogenic Differentiation of pBMSCs with or Without BMP-2

From day 21, the average osteogenic differentiation potential was about 2 for pBMSCs treated with OM and about 4 for pBMSCs with OM + BMP-2. The osteogenic differentiation potential increased to an average of 4.5 (OM) and 8.5 (OM + BMP-2) on day 28 (Figure 2, Table S1). Osteogenesis achieved by BMP-2 supplementation (OM + BMP-2) in pBMSCs was significantly increased compared to cells treated with OM alone (OM) (*p* ≤ 0.05).

### 3.3. Comparison of Osteogenic Differentiation of pASCs and pBMSCs Under Specific Inhibition from TGF-β, BMP, and FGF Signaling

#### 3.3.1. Impact of TGF-β Signaling on Osteogenic Differentiation of pASCs and pBMSCs

**Figure 3:**
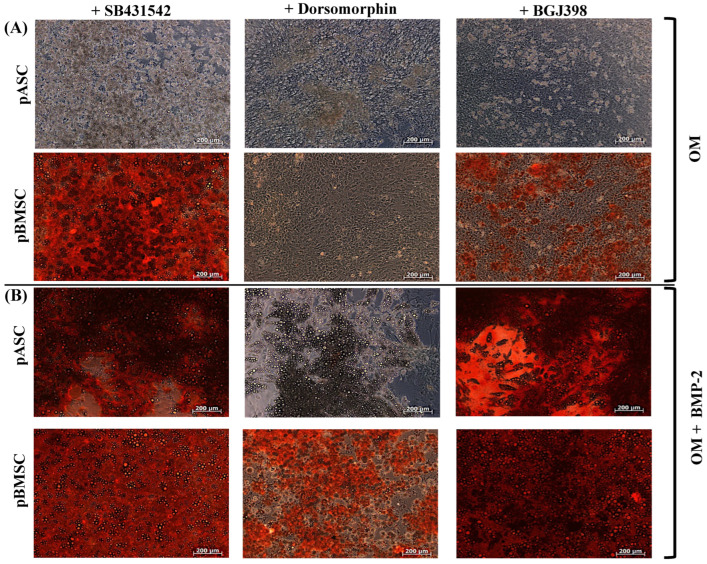

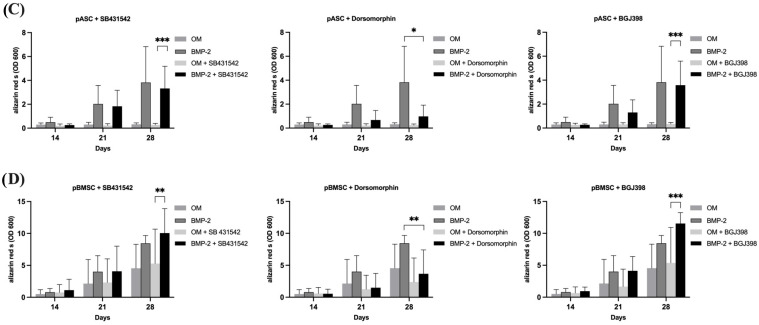
Effects of co-inhibition of TGF-B, BMP, or FGF signaling on osteogenic differentiation of pASC and pBMSC after facultative BMP-2 supplementation. Exemplary microscopic representation of alizarin red S staining in pASC and pBMSC after 28 days of differentiation with (**A**) OM and (**B**) addition of BMP-2 (450 ng/mL) with the inhibitors SB431542, dorsomorphin, and BGJ398 (scale bar = 200 um). (**C**,**D**) Photomercial quantification of the osseous differentiation of pASC and pBMSC cultured with OM +/− BMP-2 for up to 28 days in addition to the inhibitors SB431542, dorsomorphin, and BGJ398. Significant differences are marked (* *p* ≤ 0.05, ** *p* ≤ 0.01, *** *p* ≤ 0.001; pASC *n* = 6; pBMSC *n* = 4; BMP-2 [450 ng/mL]; SB431542 [1 µM]; dorsomorphin [0.5 µM]; BGJ398 [0.5 µM]).

To investigate the impact of TGF-β signaling on osteogenic differentiation, pASCs and pBMSCs were co-incubated with reversible SB431542 and additionally treated with BMP-2. SB431542 is a selective inhibitor of TGF-β signaling, which, respectively, inhibits activin receptor-like kinase (ALK) receptors ALK5, ALK4, and ALK7 [39]. It also inhibits TGF-β-induced gene transcription and expression. MSC co-incubated with BMP-2 showed a significantly increased osteogenic differentiation capacity from day 21, both with and without inhibition of the TGF-β signaling pathway in pASCs and pBMSCs (Figure 3, Table S2). In the late phase (day 28), inhibition of TGF-β signaling tended to inhibit the osteogenic differentiation of pASCs but was not significant (*p* = 0.98). The inhibition of TGF-β signaling in pBMSCs instead led to qualitatively better osteogenic differentiation in the late phase in the pBMSCs in both groups (OM +/− BMP-2) (Figure 3, Table S2). When SB431542 was applied, matrix mineralization was also visible for both pASCs and pBMSCs under BMP-2 supplementation. (Figure 3A,B).

#### 3.3.2. Impact of BMP Signaling on Osteogenic Differentiation of pASCs and pBMSCs

To investigate BMP signaling, pASCs and pBMSCs were, respectively, co-incubated with dorsomorphin. Dorsomorphin irreversibly and selectively inhibits BMPR-I, ALK2, ALK3, and ALK6 [35]. As expected, the inhibition of BMP signaling with dorsomorphin resulted in significantly reduced osteogenesis in pASCs in OM + BMP-2 after 21 and 28 days compared to the OM + BMP-2 group without inhibition (*p* ≤ 0.05) (Figure 3A,C). The same observation was made for pBMSCs, where the inhibition of BMP signaling with dorsomorphin significantly reduced osteogenesis with and without BMP-2 (*p* ≤ 0.01) (Figure 3B,D).

#### 3.3.3. Impact of FGF Signaling on Osteogenic Differentiation of pASCs and pBMSCs

To investigate the effect of FGF signaling, pASCs and pBMSCs with or without BMP-2 were co-incubated with BGJ398. BGJ398 is a reversible FGFR 1-3 inhibitor [40,41,42]. In the late phase (day 28), the inhibition of FGF signaling resulted in a comparable osteogenic differentiation between pASCs (OM + BMP-2), with and without the incubation of BGJ398 (Figure 3A,C). The inhibition of FGF signaling with BGJ398 did not seem to affect the successful osteogenic differentiation of the pASCs. In contrast, the inhibition of FGF signaling in pBMSCs led to a significant qualitative improvement (*p* ≤ 0.001) in the late phase of osseous differentiation compared to the pBMSCs without inhibition, resulting in the highest value for OM + BMP-2 + BGJ398 (Figure 3B,D). This effect was more pronounced in the groups with BMP-2 supplementation than in the groups with OM alone.

#### 3.3.4. Impact of the Simultaneous Inhibition of TGF-β and BMP Signaling on Osteogenic Differentiation of pASCs and pBMSCs

**Figure 4:**
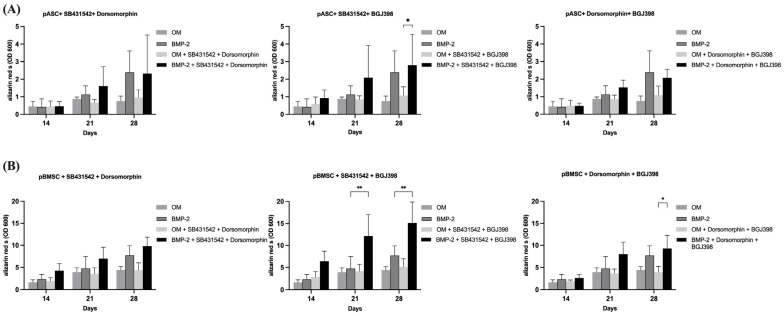
The effects of inhibitor combinations on osteogenic differentiation of pASC and pBMSC. Photometrical quantification of osseous differentiation of (**A**) pASCs and (**B**) pBMSCs. Cells were cultured in OM +/− BMP-2 with combined addition of respective inhibitors SB431542, dorsomorphin, and BGJ398. Combined utilization of SB431542 (TGF-inhibitor) and BGJ398 (FGF-inhibitor) showed the highest results in both pASC and pBMSC. Significant differences are marked (* *p* ≤ 0.05, ** *p* ≤ 0.01; pASC *n* = 6; pBMSC *n* = 4; BMP-2 [450 ng/mL]; SB431542 [1 µM]; dorsomorphin [0.5 µM]; BGJ398 [0.5 µM].

To investigate the simultaneous inhibition of TGF-β and BMP signaling, pASCs and pBMSCs were co-incubated with SB431542 and dorsomorphin and treated with OM +/− BMP-2. The pASCs treated with OM + BMP-2 and co-incubated with both SB431542 and dorsomorphin surprisingly showed positive osteogenic differentiation with a similar course in quality as those without inhibitors (Figure 4A, Table S3). Likewise, surprisingly, for pBMSCs treated with OM + BMP-2, osteogenic differentiation even tended to be improved under the inhibition of TGF-β and BMP signaling compared to those with no inhibitor used (Figure 4B, Table S3). The additional inhibition of TGF-β signaling in pBMSCs and pASCs abolished the inhibition of osteogenic differentiation using dorsomorphin in both groups (OM +/− BMP-2).

#### 3.3.5. Impact of Simultaneous Inhibition of TGF-β and FGF Signaling on Osteogenic Differentiation of pASCs and pBMSCs

**Figure 5:**
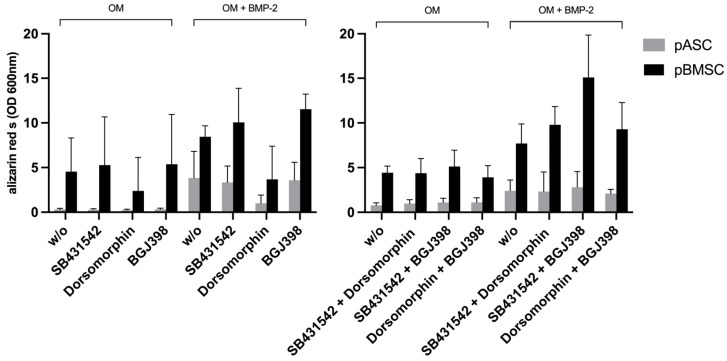
Overview of osteogenic differentiation of pASC vs. pBMSC on day 28. Overview of a comparison of pASCs and pBMSCs on day 28 of osseous differentiation. It was shown that the use of SB431542 and BGJ398 in combination led to the strongest osseous differentiation, while the inhibitory effect of dorsomorphin can be reversed by co-inhibition of BGJ398 or SB431542.

To investigate the effects of TGF-β and FGF signaling on pASCs and pBMSCs, MSCs were co-incubated with SB431542 and BGJ398. Already in the early phase after 14 days of co-inhibition of TGF-β and FGF signaling, the pASCs showed improved osteogenic differentiation (*p* ≤ 0.05), culminating in the qualitatively highest results after 28 days (Figure 4, Table S3). Likewise, the pBMSCs showed significantly higher values (OD 600 of 6.4) of osteogenic differentiation in the OM + BMP-2 group when inhibitors were applied compared to those without inhibitors (OD 600 of 2.8). In the later phase, from day 21 to 28, the co-inhibition of TGF-β and FGF signaling showed increasing improvement in osteogenic differentiation in the OM + BMP-2 group, with its highest value on day 28 (*p* ≤ 0.001). In contrast, the utilization of inhibitors SB431542 and BGJ398 without BMP-2 supplementation showed no improvement in the osteogenic differentiation of the pBMSCs and resulted in a similar course as was seen for the pBMSCs differentiated using OM only (Figure 4B, Table S3). The simultaneous inhibition of TGF-β and FGF signaling resulted in the highest values of osteogenic differentiation for the pASCs and pBMSCs co-incubated with BMP-2 (*p* ≤ 0.001) (Figure 5).

#### 3.3.6. Impact of Simultaneous Inhibition of FGF and BMP Signaling on Osteogenic Differentiation of pASCs and pBMSCs

The investigation of BMP and FGF signaling was performed through co-incubation with dorsomorphin and BGJ398. On day 28, the inhibition of BMP-2 and FGF signaling in pASCs additionally differentiated with BMP-2 led to qualitative osteogenic differentiation, which tended to be lower than without inhibition (Figure 4, Table S3). Also, for pBMSCs, the co-inhibition of FGF and BMP signaling resulted in positive osteogenic differentiation. When the pBMSCs were additionally differentiated with BMP-2 (OM + BMP-2), and BMP and FGF signaling were inhibited after 28 days, the osteogenic differentiation tended to be higher than without inhibitor use (Figure 4, Table S3). Surprisingly, the additional inhibition of FGF signaling also mitigated the inhibition of osteogenic differentiation with dorsomorphin in both groups (OM +/− BMP-2) of pBMSCs and in pASCs with BMP-2 supplementation.

### 3.4. Evaluation of Receptor Expression in pASCs in the Course of Osteogenic Differentiation

**Figure 6:**
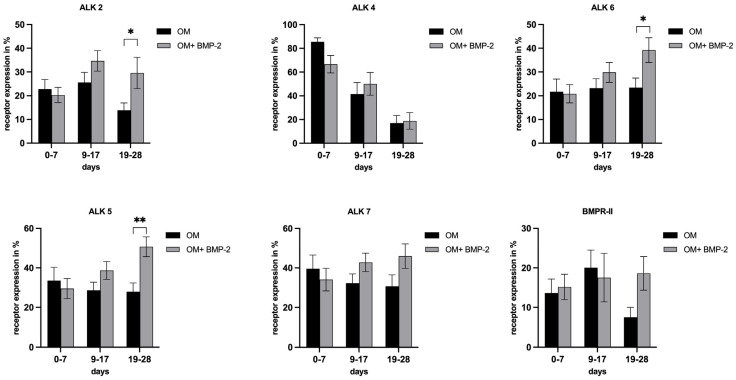
Receptor expressions of ALK2, ALK4, ALK6, ALK5, ALK7, BMPR-II in pASC. Grouped representation of the respective receptor expression in the course of osteogenic differentiation (OM +/− BMP-2). From day 19, there is a significant induction of ALK 2, ALK 6, and ALK 5 with the addition of BMP-2. BMPR-II expression in the OM group decreased in OM and tended to stay increased under BMP-2 supplementation from day 19, but was not considered significant (* *p* ≤ 0.05, ** *p* ≤ 0.01; *n* = 6, BMP-2 450 ng/mL).

To determine whether successful differentiation of pASCs is associated with modulation of the corresponding receptor expressions of TGF-β (ALK4, ALK5, and ALK7) and BMP signaling (ALK2 and ALK6), these were analyzed over the course of osseous differentiation (±BMP-2). As previously described, osteogenic differentiation of stromal cells is a gradual process that progresses at different rates. As a result, the findings were categorized into early (0–7 days), mid (7–14 days), and late (14–28 days) differentiation phases as described before [43,44]. Accordingly, from day 19, a significant increase in ALK2 expression with the addition of BMP-2 was detected (*p* < 0.05) (Figure 6). With the additional treatment with BMP-2 (OM + BMP-2), the expression of ALK6 increased significantly in the pASCs from day 19 to 28 (*p* < 0.05) (Figure 6). Expression of the ALK4 receptor decreased comparably in the pASCs with OM +/− BMP-2 over the course of osteogenic differentiation (Figure 6). Under incubation with BMP-2, ALK5 expression showed a steady increase, from day 19 onward, and pASCs treated additionally with BMP-2 showed a significant induction of ALK5 expression in comparison to the OM group (*p* < 0.01) (Figure 6). The expression of ALK7 hardly changed in the course of 28 days of osteogenic differentiation with and without BMP-2 (OM +/− BMP-2) (Figure 6). After 19 to 28 days, BMPR-II expression in the OM group decreased (Figure 6).

### 3.5. Analysis of Key Protein Expressions of TGF-β, BMP, Wnt, MAPK, and FGFR Signaling in pASCs

**Figure 7:**
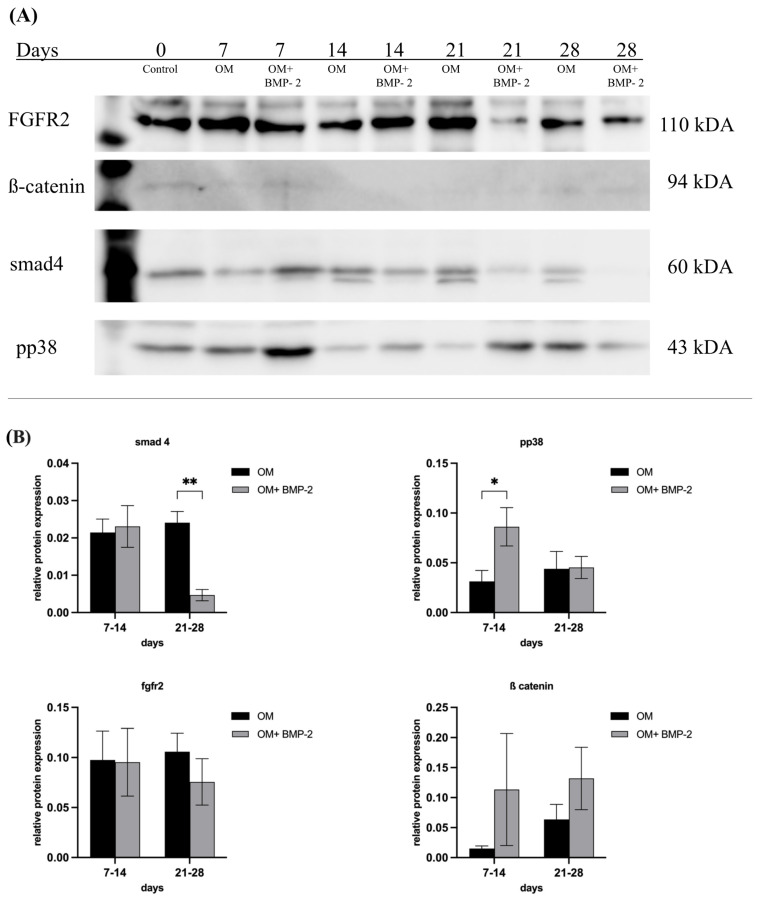
Key protein expression of smad4, pp38, β-catenin, fgfr2 in pASC. (**A**) Western blot of key proteins. (**B**) Grouped representation of the relative protein expression of key proteins of BMP-, Wnt-, MAPK, and FGFR signaling under incubation in OM and under addition of BMP-2 (450 ng/mL) to OM. BMP-2 supplementation led to a significant increase of pp38 (* *p* ≤ 0.05) and tendency of β-catenin. Over the course, a significant decrease of smad4 under BMP-2 supplementation was seen (** *p* ≤ 0.01). *n* = 6.

To evaluate the effects of BMP-2 on the BMP, Wnt, MAPK, and FGFR signaling pathways, in terms of successful osteogenic differentiation, the expressions of the corresponding transcription factors were analyzed over a period of 28 days. Time frame 7–14 days covers the early phase of osteogenetic differentiation process, as this is described as the phase of early cell differentiation during which MSCs start expressing osteogenic markers secreted by early osteoblasts (matrix maturation phase). This is followed by a mineralization phase (days from 14 to 28) where a high expression of osteocalcin and osteopontin, secreted by late osteoblasts, is observed, and accompanied by calcium and phosphate deposition [43,44]. Over the course of successful osteogenic differentiation of pASCs treated with BMP-2, TGF-β, and, subsequently, BMP signaling, was significantly downregulated. Smad4 decreased significantly during the late phase of differentiation under the influence of BMP-2 (*p* ≤ 0.01) (Figure 7). The relative protein expression of pp38, representing a key protein of MAPK signaling, was significantly higher in the early phase from day 7 to 14 when BMP-2 was used (*p* ≤ 0.05) (Figure 7). For ß-catenin, representing a key transcription factor of Wnt signaling, a higher expression within days 7–14 for the OM + BMP-2 group was detected (Figure 7). The expression of FGFR2 was increased over the entire course of 28 days but did not show significant differences between the two test groups (Figure 7).

## 4. Discussion

Ideal bone graft substitutes should be biocompatible, bioresorbable, osteoconductive, osteoinductive, structurally similar to natural bone, and easy to use. Referring to this, several previous studies have confirmed that combining MSCs with a supporting scaffold is a promising therapeutic alternative for bone repair and regeneration [5,10,11,12,15,45,46]. BMSCs and ASCs have lately been the subject of more comprehensive research promoting their highly sufficient osteogenic differentiation and their considerable potential as a regenerative tool for treating critical-size bone defects [13,15,47,48]. To justify the most efficient and standardized use of ASCs and BMSCs for clinical application, comprehensive preclinical research is required to elucidate the crucial mechanisms by which these cells regenerate bone. For instance, the potential side effects of employing MSCs in bone healing still remain unclear [35,49]. Pig is the most representative large animal model for human bone regeneration processes with regard to bone anatomy, morphology, bone regeneration rates (1.2–1.5 μm/d for minipigs vs. 1.0–1.5 μm/d for humans), remodeling, and mineral density [7,20,49]. To ensure successful translation, a better understanding of the molecular osteogenic differentiation processes of pASCs and pBMSCs is necessary in order to reliably interpret certain findings in future in vivo investigations. It was shown that, in contrast to human ASCs, the osteogenic differentiation of pASCs is inhibited without the addition of BMP-2 [21]. Even in human patients, bone regeneration can be inhibited and may require specialized therapy or the addition of BMP-2 [12,50]. Previous studies on human bone healing found that aberrations in BMP signaling and an imbalance in the local presence of BMP and BMP inhibitors may switch the direction toward healing or non-healing of a fracture [51]. Understanding the molecular key processes that drive BMP-2-induced differentiation of pASCs will provide detailed insights to enhance the effectiveness of MSC-based regenerative treatments, improving the outcomes of bone repair therapies. If the significance of the interlocking signaling cascades is understood, inductions and inhibitions of certain regulatory proteins could positively influence the osteogenic differentiation of ASCs and BMSCs also in human systems. These insights are essential for selecting the most suitable and well-prepared MSCs in combination with an appropriate scaffold for future in vivo studies. This knowledge can be leveraged to improve human therapies for non-healing bone defects. By optimizing the cell–scaffold combinations, we can enhance the effectiveness of regenerative treatments for bone repair in clinical settings.

In this study, the qualitative and quantitative osteogenic differentiation potentials of pASCs were found to be inferior to those of pBMSCs. To elucidate the differences in the molecular mechanisms between pASCs and pBMSCs, TGF-β signaling was analyzed in detail during the osteogenic differentiation of pASCs and pBMSCs. For this purpose, SB431542, an inhibitor of the activin-induced phosphorylation of Smad2 mediated by ALK4, ALK5, and ALK7, was utilized. This approach helps to dissect the specific signaling pathways involved and understand how they contribute to the osteogenic differentiation processes in these MSC types [36]. With BMP-2 supplementation, SB431542 in pASCs caused no significant difference in the osteogenesis over the course of 28 days (Figure 3). In contrast, the osteogenic differentiation of pBMSCs with and without BMP-2 was further enhanced by the inhibition of TGF-β signaling (Figure 3). TGF-β induces the recruitment of additional MSCs in critical bone defects in vivo. Depending on the cell type, TGF-β activates proliferation, differentiation, and apoptosis both via ALK4- and ALK-5-mediated phosphorylation of Smad2 and via the p38 MAPK osteoblast differentiation [24,52,53,54]. Previous studies have indicated that osteolytic bone remodeling releases TGF-β and activates the pathway, deteriorating the MSCs. The over-activation of TGF-β in vitro can dramatically suppress the maturation of hematopoietic progenitors, while the pharmacologic inhibition of TGF-β signaling has been demonstrated to restore hindered hematopoiesis under pathological states of hematopoietic stem cells in vitro and in vivo, revealing a regulatory function of TGF-β [55]. In line with this, the inhibition of TGF-β signaling showed an enhancement in osteogenesis in pBMSCs with BMP-2 supplementation. The fact that TGF-β and BMP signaling compete for Smad4 [56] suggests that, due to the inhibition of TGF-β signaling, the supplementation of BMP-2 leads to an improvement in the differentiation of pBMSCs [32]. Since inhibiting the TGF-β signaling did not have an enhancing effect on the pASCs with BMP-2, it can be assumed that the TGF-β signaling pathway plays only a minor role in the osteogenic differentiation of pASC (Figure 3).

For the inhibition of BMP signaling, dorsomorphin was chosen, as it inhibits the phosphorylation of Smad1, 5, and 8 by BMP as well as the constitutively activated BMP receptors ALK1, ALK2, ALK3, and ALK6, without affecting the TGF-β1- or activin-induced phosphorylation of Smad2 and Smad3 [57]. As expected, incubation with dorsomorphin led to a retardation of osteogenesis in all groups regardless of BMP-2 supplementation. This underpins the necessity of BMP signaling for enabling osteogenesis in pBMSCs and especially in pASCs. Furthermore, it can be concluded that pBMSCs, in contrast to pASCs, appear to produce enough BMP-2 endogenously to achieve osteogenic differentiation in OM.

BGJ398 is an inhibitor of ligand-induced autophosphorylation of FGF receptor tyrosine kinases 1, 2, and 3, preventing further phosphorylation steps, which regulate proliferation and differentiation processes [58]. The early application of FGF-2 during osteogenic differentiation accelerates osteogenesis by increasing the expressions of TGF-β and BMP-2 [59]. Under BMP-2 supplementation with the addition of BGJ398, the pASCs did not show any significant changes in osteogenesis compared to the pASCs without FGF inhibition (Figure 3). The pBMSCs with BMP-2 supplementation showed a tendency toward increased osteogenesis when the FGF signaling was inhibited compared to the pBMSCs without inhibition (Figure 3). Thus, the functions of FGF receptor tyrosine kinases 1, 2, and 3 might not have an essential importance for the osteogenic differentiation of pASCs since their inhibition did not reduce the osteogenic differentiation potential under BMP-2 supplementation. There appeared to be an impact on the osteogenesis of pBMSCs by the inhibition of FGF signaling, as indicated by improved late osteogenesis. Systemically administered FGF showed anabolic effects on bone formation in animals, while continuous treatment with FGF inhibited osteogenic differentiation in vitro [28]. These apparently contradictory outcomes indicate that the effect of FGF may depend on the differentiation stage of the osteoblasts and, regarding the findings of our study, seems to be specific to the cell type. Previous studies have found that sequential treatment with FGF-2 followed by BMP-2 enhances osteogenic differentiation in vitro [26]. A synergistic effect on osteogenic differentiation through FGF and BMP signaling appears to have a biphasic effect on osteoinductive activity, while it increases with low doses of FGF-2 and decreases with high doses of FGF-2 [27,60]. In this study, the inhibition of FGF signaling did not reduce the osteogenic differentiation of pASCs or pBMSCs. According to previous studies on MSCs, FGF-2 appears to be a positive regulator of osteoprogenitor cells and a negative regulator of the osteoblast differentiation of ASCs [28]. This might explain the improved osteogenesis in the late phase in pBMSCs under BMP-2 and the inhibition of FGF. For further evaluation, pMSCs were differentiated under the combined inhibition of two of the investigated signaling pathways.

While the inhibition of BMP signaling led to a significant reduction in the osteogenic differentiation of pASCs and pBMSCs, this retarded differentiation by dorsomorphin was reversed by the simultaneous inhibition of TGF-β signaling with SB431542 under BMP-2 supplementation. Moreover, it resulted in an overall improvement in the osteogenesis of pBMSCs (Figure 4). The inhibition of TGF-β signaling might provide a competitive advantage to BMP-2 supplementation, which makes BMP signaling more effective, despite the use of dorsomorphin, because dorsomorphin is a reversible inhibitor. This is possibly due to the known crosstalk between the two signaling pathways [61], because BMP and TGF-β signaling have been described to negatively regulate one another through crosstalk involving Smad proteins [62]. This assumption is corroborated by the fact that in the pBMSCs without BMP-2 supplementation, the osteogenic differentiation did not increase when TGF-β and BMP signaling were inhibited. BMP-2 expression is induced during the early phase of fracture healing, whereas TGF-β expression increases later, suggesting that a temporal expression pattern of these growth factors is of overriding importance [34,63]. Similarly, under BMP-2 administration, the simultaneous inhibition of FGF and BMP signaling in both pASCs and pBMSCs still resulted in osteogenic differentiation. In contrast, inhibiting BMP signaling alone completely prevented osteogenesis (Figure 4*).* This may be due to the fact that dorsomorphin is a reversible inhibitor of BMP signaling, and the inhibition of FGF seemingly provides a competitive advantage to BMP-2 supplementation over inhibition using dorsomorphin. This competitive dynamic allows BMP-2 to effectively promote osteogenic differentiation even in the presence of dorsomorphin. This suggests a complex interplay between these pathways, where BMP-2 can still promote osteogenic differentiation under certain inhibitory conditions, emphasizing its dominant role in this process. Previous studies have found that the sequential addition of FGF-2 followed by BMP-2 in a murine model led to the best possible osteogenic differentiation, while the combined addition of osteoinductive growth factors did not necessarily result in increased osteogenesis through induced signaling [34,64]. The function of FGF-2 in osteoblastic differentiation depends on the cell type, maturation stage, and its concentration. Stimulating effects on osteoprogenitor engagement were found in early differentiation, whereas an inhibitory effect was found on terminal osteoblastic differentiation and mineralization by preventing the upregulation of BMPs and BMPRs in human MSCs [65,66]. Exogenous FGF-2 antagonizes the upregulation of BMPR-IB gene expression, indicating that higher threshold levels of BMPR-IB may play a crucial role in counteracting the inhibitory role of FGF-2, promoting the osteogenic differentiation of ASCs [67]. Conclusively, to further break down the impact of TGF-β and FGF signaling, sequential inhibition or induction would be a valuable approach to evaluate their regulatory mechanisms decisive for osteogenesis, which cannot be detected through continuous pathway inhibition. When TGF-β and FGF signaling were simultaneously inhibited (SB431542 + BGJ398) in pASCs and pBMSCs under BMP-2 supplementation, their osteogenic differentiation was increased in contrast to that of the cells without inhibitors. This resulted in the highest values of osteogenic differentiation of both the pASCs and pBMSCs (Figure 4). Both TGF-β and FGF-2 have been proven to be inducers of the proliferation of osteoblasts, but they reduce the expression of alkaline phosphatase, and thus, mineralization in vitro [68]. It appears that the inhibition of TGF-β signaling as a competitor for Smad4 and the inhibition of FGF signaling as a negative regulator of osteoblasts and BMPR upregulation [28,56,62,69] lead to an advantage of BMP-2 signaling and amplification of the effect of BMP-2 supplementation. This underlines that BMP signaling is of the highest importance for osteogenic differentiation and that BMP-2 supplementation, therefore, largely determines the osteogenic differentiation of pBMSCs and especially of pASCs. In order to be able to make reliable statements about the osteogenic differentiation potential of MSCs for in vivo application, further study is recommended.

### pp38, a Regulatory Signaling for BMP-2 Induced Osteogenic Differentiation in pASC?

Receptors associated with BMP (activin-like kinases ALK2, ALK3, and ALK6 and BMP receptors BMPR-I and BMPR-II) and TGF-β signaling (ALK4, ALK5, and ALK7) were analyzed to investigate if improved osteogenic differentiation, or differentiation made possible in the first place, is associated with the modulation of respective receptor expression in pASC (OM + BMP-2). The supplementation of BMP-2 significantly increased the expressions of ALK2 and ALK6 in the pASCs. Yet, there were significant increases in their expressions in the late phase (days 19–28) of osteogenic differentiation compared to the pASCs without BMP-2 supplementation, which failed to differentiate osteogenically (Figure 6). This late receptor upregulation is consistent with the results of previous studies that considered the Smad-induced regulation of ALK2 as causative, assuming a similar mechanism for ALK6 [50,65]. Characteristic TGF-β receptors ALK4 and ALK7 were not significantly influenced by BMP-2 supplementation in comparison to pASCs in OM only, but a significant late induction (days 19–28) of ALK5 under BMP-2 influence was observed (Figure 6). In a previous study, it was demonstrated that the binding of BMP-2 to heteromeric receptor complexes consisting of BMPR-I, TGF-β receptor I, and TGF-β receptor II resulted in crosstalk with TGF-β signaling. Under the treatment of BMP-2, complex formations among the BMP-binding TGF-β superfamily receptors (among others, consisting of BMPR-II/ALK5, ALK5/ALK7, and ALK3/ALK5) lead to the phosphorylation and activation of the TGF-β targets Smad2 and Smad3, thus documenting an interaction of BMP with TGF-β signaling [61]. This crosstalk with TGF-β signaling via ALK5 might be an explanation of the late induction of ALK5 from the addition of BMP-2 in pASCs. The late upregulation of BMPR II and ALK 2, 6, and 5 due to BMP-2 supplementation appears to be a crucial step that modulates the osteogenic differentiation of pASCs. The expression of the key protein Smad4 was investigated to examine BMP and TGF-β signaling. Smad4 is a common-mediator SMAD (co-SMAD) essential for both signaling cascades. It facilitates the nuclear translocation of receptor-regulated SMAD (R-SMAD) complexes, namely Smad1/5/8 in BMP signaling and Smad2/3 in TGF-β signaling, by forming transcriptionally active complexes. Notably, BMP and TGF-β pathways compete for Smad4, which acts as a central integrator of both signaling networks. Smad4 has a mediating role between bone formation and resorption, since the complex regulates gene transcription via Dlx5 and Runx2 by binding to SBEs (Smad binding elements) [24,56,70]. BMP-2 supplementation did not affect Smad4 expression in pASCs during the early differentiation phase, but led to significant downregulation of Smad4 in the late phase from day 21 (Figure 7). This late-phase inhibition of Smad4 appears to be critical for the progression of osteogenic differentiation, particularly for the transition from osteoblasts to osteocytes. Previous studies found that the ablation of Smad4 enhanced proliferative responses to canonical Wnt signaling through interactions with β-catenin and balanced the pro-mitogenic and pro-mineralizing actions of β-catenin in osteoblasts [71]. It was proposed that Smad4 and Tcf/Lef (T-cell transcription factor/lymphocyte enhancer factor) transcription complexes compete for β-catenin, restraining Wnt-dependent proliferative signals while favoring the matrix-synthesizing activity of osteoblasts in the late phase [71]. This balancing purpose of Smad4 might be an explanation why late downregulation of Smad4 is necessary to allow a proper osteogenic differentiation. Still, the signaling by which the inhibition of Smad4 is mediated in the late phase could not be conclusively clarified in this study. We acknowledge assessing phosphorylated SMAD1/5/9 as a more direct readout of BMP pathway activity, but the focus on Smad4 in this study was intended to capture the integrative and regulatory roles of both BMP and TGF-β pathways. Given its central role in mediating transcriptional activity across both pathways, Smad4 provides insight into the downstream convergence of signaling mechanisms that govern osteogenic commitment. Nevertheless, future studies should aim to include phospho-SMAD profiling to dissect the pathway-specific dynamics more precisely. To investigate canonical Wnt signaling, the expression of β-catenin in pASCs under BMP-2 supplementation was measured, since canonical Wnt signaling is essential for MSC differentiation to osteoblast-lineage cells [72]. Due to the complex formation of R-Smads, β-catenin, and Tcf/Lef, BMP signaling and Wnt signaling synergistically regulate the transcription factors of the same target genes in osteoblasts [31,35,71,73]. In this study, β-catenin showed steady upregulation under BMP-2 supplementation compared to pASCs in OM only (Figure 7). Thus, the expression of β-catenin induced by the addition of BMP-2 plays a basic, key role in the osteogenic differentiation of pASCs (Figure 7). This is consistent with previous studies, which found that Wnt signaling is stimulated by BMP activity, creating β-catenin/TCF/LEF/RUNX2 complexes [74]. In cooperation with Wnt/β-catenin, FGF signaling activates the expression of transcription factor Runx2, regulating the expression of osteoblast-specific genes [75,76]. To evaluate FGF signaling, the expression of FGFR2 in pASCs under the influence of BMP-2 was examined, but no significant effect on the expression of FGFR2 over the entire period was detected (Figure 7). Thus, FGF signaling does not appear to be a crucial pathway in reversing the inhibition of the osteogenic differentiation of pASCs under BMP-2 supplementation. In our study, the expression of pp38 was investigated as an activated key protein for p38 MAPK signaling. p38 MAPK signaling is significantly involved in the induction of osteoblast differentiation via the regulation of the transcription factors Runx2, Dlx5, and Osterix [24,52]. Previous studies have found a corresponding accelerating effect of pp38 on osteogenic differentiation by its upregulation in the early phase [77,78]. In this study, it was found that pp38 was significantly higher in pASCs under the influence of BMP-2 in the early phase up to day 14 (Figure 7). Conclusively, BMP-2 supplementation promoted the activation of underlying p38 MAPK signaling in the early phase of osteogenic differentiation, which might be imperative for the induction of the osteogenic differentiation of pASCs. We assume that the osteogenic differentiation of pASCs is mediated by BMP-2-induced MAPK signaling through the activation of TAK1 and TAB1. Our assumption is supported by previous protein analysis on human ASCs, which showed elevated pp38 expression in hASCs [35]. Previous studies have indicated that Smad-independent pp38 (MAPK) activation through BMP-2 supplementation is necessary for the acquisition of the osteoblast phenotype using pluripotent C2C12 cells [77]. BMP-2 supplementation leads to the synergistic early activation of MAPK and Wnt signaling to induce the osteogenic differentiation of pASCs. Wnt signaling leads to β-catenin accumulation, enabling the early and Smad-independent transcription of target genes. Since no investigations on this matter were conducted in this study, we hypothesize that BMP-2 in pASC facilitates osteogenic differentiation through early Smad-independent p38 induction. This hypothesis needs to be confirmed in further studies. p38 seems to be the essential participant in the transcription of factors like Runx2 and in the regulation of β-catenin accumulation by targeting β-catenin/TCF/LEF/RUNX2 complexes and further steps of Wnt signaling [52,74], whereas pASCs appear to be unable to initiate this early activation of p38 and Wnt if BMP-2 is not supplemented. Prior studies have suggested that variation of key osteogenic transcription factors like RUNX2 and SP7, as well as autocrine BMP signaling may be associated with differences in osteogenic differnetiation potential [79]. These aspects are certainly worth exploring in future mechanistic studies. Phosphorylation of p38 is pivotal step in osteogenesis, promoting cell differentiation and enhancing the expression of osteogenic markers such as alkaline phosphatase and osteocalcin [52]. It must be acknowledged that assessment of the precise activation status of p38 requires a pp38/p38 ratio, since elevated pp38 alone can be misleading if total p38 is also increased. The pp38/p38 ratio allows definitive conclusions about signaling effectiveness, especially when baseline p38 levels differ across cell types. Previous studies have demonstrated that total p38 levels remain relatively stable under osteogenic conditions in MSCs [80]. Therefore, a comparative analysis of treatment response in cell cultures (BMP-2- induced phosphorylation of pp38) observing a marked increase in pp38 alone deemed sufficient to confirm treatment effect attributed to BMP-2 supplementation. This approach is commonly applied in preliminary analyses when the primary aim is to confirm treatment responsiveness, rather than to quantify signaling efficiency across conditions [80]. This study can confirm that an increased activation of p38 by BMP-2 supplementation is associated with successful osteogenic differentiation of pASC. Our findings confirm that BMP-2 supplementation at 450 ng/mL effectively activates p38 in pASCs and is associated with successful osteogenic differentiation. However, this study cannot definitively prove that p38 MAPK signaling is the sole pathway mediating BMP-2-induced osteogenesis. It is likely that endogenous BMP-2 levels in pASCs are insufficient to trigger adequate MAPK activation under osteogenic medium alone. To identify the key regulatory factors underlying this differentiation process, future studies employing comparative proteomics will be necessary. In addition to canonical signaling pathways such as BMP-mediated Smad activation, MAPK (p38), and Wnt/β-catenin signaling, recent evidence suggests that DNA damage itself may serve as a potent stimulus for osteogenic differentiation in mesenchymal stromal cells. Rosina et al. demonstrated that genotoxic stress can induce osteogenic commitment via activation of the DNA damage response, including p53 and ATM signaling pathways, independent of classical osteoinductive cues [79].

While our study did not specifically assess DNA damage or related stress pathways, the possible interplay between BMP-2 stimulation and cellular stress responses warrants consideration. It is conceivable that high-dose BMP-2 or prolonged culture conditions may indirectly induce DNA stress, thereby enhancing osteogenic commitment via non-canonical routes. Future studies could explore whether the activation of osteogenesis in our system involves components of the DNA damage response, particularly under conditions where canonical pathway activation appears insufficient.

## 5. Conclusions

The present study found that the osteogenic differentiation of pASCs is critically dependent on BMP-2 supplementation and observed that BMP-2 supplementation is associated with increased phosphorylation of p38 and activation of Wnt signaling in the early phase compared to non-differentiating pASCs without BMP-2. It is most likely that BMP-2 is essential for inducing a synergistic activation of p38 and ß-catenin and enables an early Smad-independent transcription of target genes, which are essential factors for initiating the osteogenesis of pASCs. Since the receptor density in the early phase of osteogenic differentiation showed no difference between the pASC groups (OM vs. OM + BMP-2), pASCs do not seem to be able to express enough endogenous BMP-2 to activate p38 MAPK and Wnt to successfully induce osteogenic differentiation. Suppression of osteogenesis through BMP inhibition can be reversed in both pASCs and pBMSCs by co-inhibiting FGF or TGF-β signaling. The simultaneous inhibition of TGF-β and FGF signaling under BMP-2 supplementation achieved the greatest possible calcium deposition in the extracellular matrix. The inhibition of TGF-β signaling as a competitor for Smad4 and the inhibition of FGF signaling’s inhibitory effect on terminal osteoblastic differentiation benefited BMP-2, serving certain crosstalk checkpoints and enhancing the effect of BMP signaling on osteogenic differentiation. Thus, our findings affirm that (endogenous) BMP-2 plays a key role in osseous differentiation of pASC, whereas both TGF-β and FGF signaling appear to play moderating roles in terms of the quality of osteogenesis in both pASC and pBMSC. Our study demonstrates that pBMSCs have significantly better potential for osteogenic differentiation than pASCs. In addition to considerations of optimal cell count and senescence related to cell type and cultivation, the fine-tuning of TGF-β, BMP, and FGF signaling is a crucial parameter in selecting and designing appropriate scaffolds for in vivo applications. Nevertheless, it should be considered that bone marrow has the disadvantage of higher donor-site morbidity, lower availability, and often insufficient yield for large bone defects compared to adipose tissue. The knowledge gained in this study may support further investigations and in vivo trials to elucidate the essential mechanisms and ensure standardized, safe, and most effective MSC-based bone therapy.

## Data Availability

The data that support the findings of this study are available from the corresponding author upon reasonable request.

## Supplementary Materials

The following supporting information can be downloaded at: https://www.mdpi.com/article/10.3390/biology14060610/s1, Figure S1: Titration of the Inhibitor SB431542; Figure S2: Titration of the Inhibitor Dorsomorphin. Figure S3: Titration of the Inhibitor BGJ398. Figure S4: FACS—Gates of pASC. Figure S5: FACS Gates of pBMSC. Table S1: OD Readings on pASCs and pBMSCs under inhibitor supplementation. Table S2: OD Readings on pASCs and pBMSCs under supplementation of inhibitor combinations. Table S3: Western blot readings of key proteins expression during osteogenic differentiation of pASCs, Readings of receptor expression during osteogenic differentiation of pASCs. Table S4: Donor Overview. Table S5: Experimental Design and Replicate Overview.

## Abbreviations

The following abbreviations are used in this manuscript:

pBMSC	porcine bone marrow-derived stromal cell
pASC	porcine adipose-derived stromal cell
TGF	transforming growth factor
FGF	fibroblast growth factor
BMP	bone morphogenic protein
MAPK	mitogen-activated protein kinase
pp38	phosphorylated p38
ALK	anaplastic lymphoma kinase
OM	osteogenic medium
DMEM	Dulbecco’s Modified Eagle Medium
LANUV	Landesamt für Natur, Umwelt und Klima Norrhein-Westfalen
