# Supplementary material for: BMP-2-Driven Osteogenesis: A Comparative Analysis of Porcine BMSCs and ASCs and the Role of TGF-β and FGF Signaling
Source: Biology (Basel). 2025 May 26;14(6):610. doi: 10.3390/biology14060610 (PMC12189446; doi:10.3390/biology14060610)
Supplement: Supplementary file 1 [file biology-14-00610-s001.zip › biology-3604629-supplementary.pdf]

Supplemental Data

BMP-2-Driven Osteogenesis: A comparative analysis of porcine BMSCs and ASCs and the role of TGF-β and FGF Signaling

Titration of inhibitors SB431542, Dorsomorphin, BGJ398 to determine the inhibitor concentration.

Treatment of pASCs with increasing concentrations of SB431542 for up to 7 days did not result in a significant reduction in cell viability compared to untreated controls. Notably, viability was slightly increased at days 2 and 5 with higher inhibitor concentrations, while a general decline in viability was observed across all groups by day 7 (Figure S1). Based on these findings, 1 µM SB431542 was selected for use in subsequent osteogenic differentiation assays. Dorsomorphin, an inhibitor of the BMP signaling pathway, was applied to pASCs in various concentrations for 24 hours. Cell viability was assessed using the CellTiter-Blue® assay, revealing a dose-dependent decrease in viability at concentrations above 1 µM, and a significant reduction also at 0.2 µM compared to untreated controls. Cells treated with 0.5 µM Dorsomorphin showed no significant reduction in viability, and this concentration was therefore used for subsequent osteogenic differentiation (Figure S2). To evaluate an appropriate concentration of BGJ398, an inhibitor of the FGF signaling pathway, pASCs were treated with varying concentrations for 2, 5, and 7 days, and cell viability was assessed using the CellTiter-Blue® assay. No significant reduction in cell viability was observed at any time point compared to the untreated control, and 500 nM BGJ398 even showed a slight increase in viability after 2 days. Based on these results, a concentration of 500 nM was selected for subsequent osteogenic differentiation experiments (Figure S3).

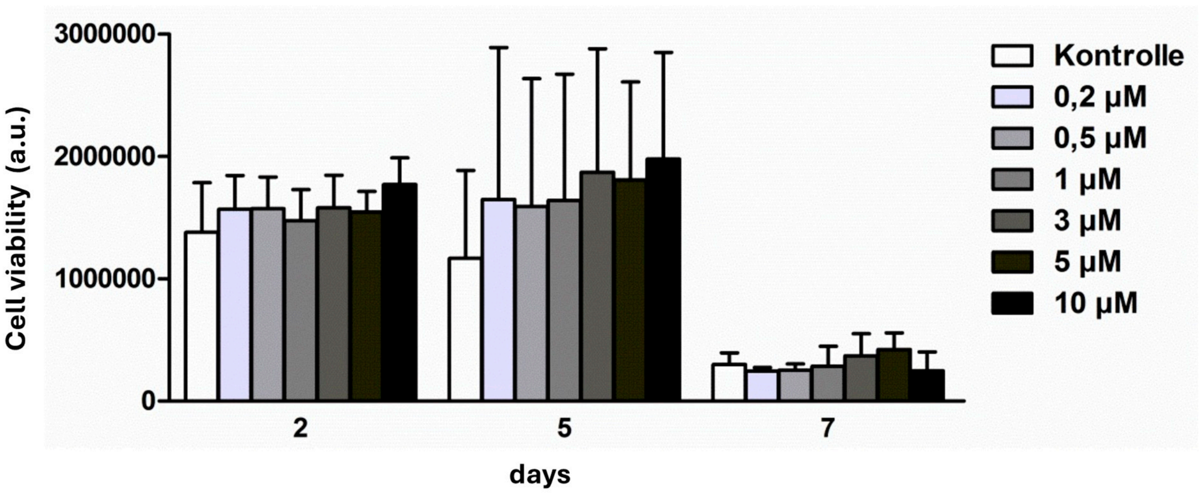

Figure S1: Titration of the Inhibitor SB431542
pASCs were incubated with various concentrations of the inhibitor SB431542 for 2, 5, and 7 days. Subsequently, cell viability was assessed using the CellTiter-Blue® cell viability assay. (a.u. = arbitrary units); n = 3

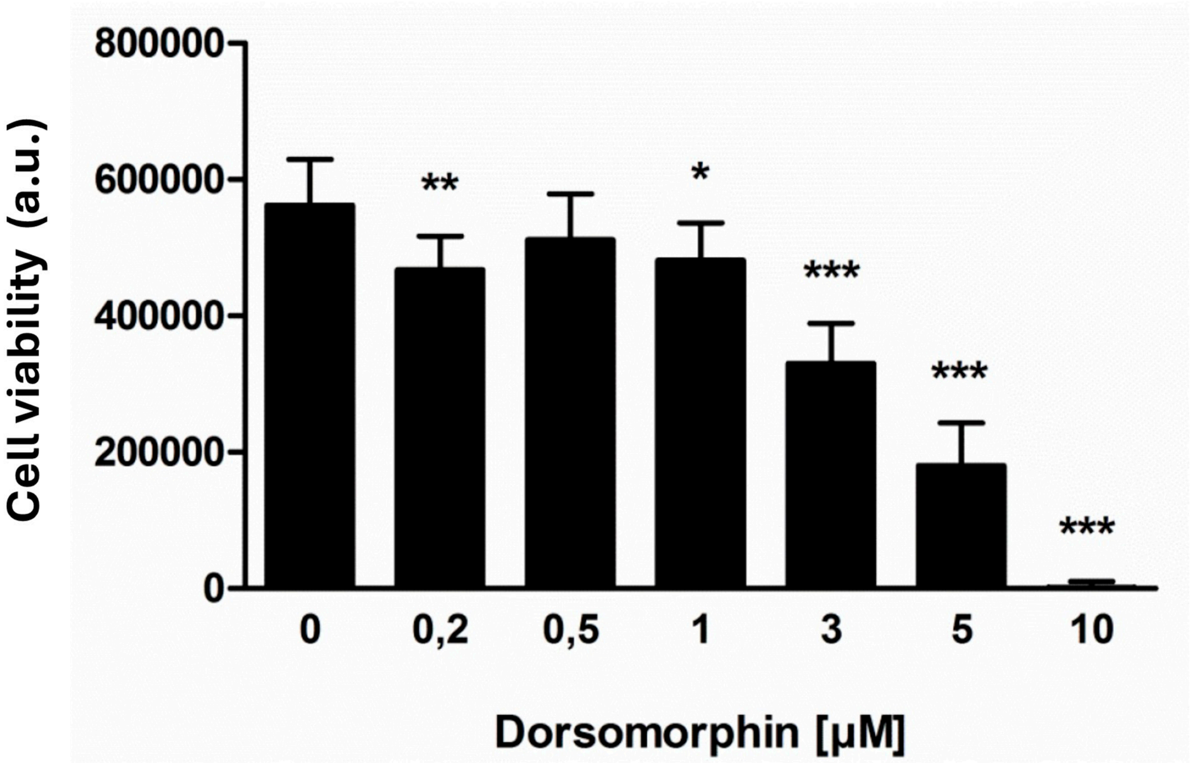

Figure S2: Titration of the inhibitor Dorsomorphin
pASCs were incubated with varying concentrations of the inhibitor Dorsomorphin for 24 hours. Subsequently, cell viability was assessed using the CellTiter-Blue® cell viability assay. Significant results are relative to the control (0 µM Dorsomorphin). (a.u. = arbitrary units); n = 3

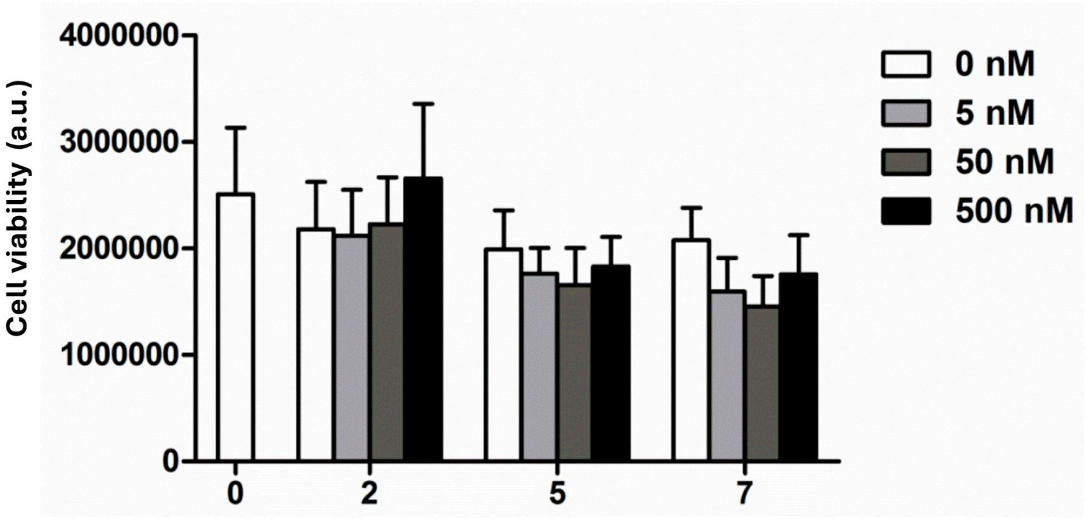

Figure S3: Titration of the inhibitor BGJ398
pASCs were incubated with various concentrations of the inhibitor BGJ398 for 2, 5, and 7 days. Subsequently, cell viability was assessed using the CellTiter-Blue® Cell Viability Assay. (a.u. = arbitrary units); n = 3

FACS- Analysis

As follows find representative dot plots showing the expression of MSC surface markers in pASCs and pBMSCs after staining with fluorochrome-conjugated antibodies. Cells were analyzed using dual-channel flow cytometry (FL1-H and FL4-H). Gating was based on FSC/SSC to exclude debris and aggregates, followed by singlet gating (FSC-A vs. FSC-H). Quadrants were defined using FMO and unstained controls.
The lower left (LL) quadrant represents marker-negative cells, while the lower right (LR) and upper left (UL) quadrants indicate cells positive for the respective marker on FL1 or FL4 channels. Double-positive cells are located in the upper right (UR) quadrant. pASCs and pBMSCs were positive for CD29, CD90, and CD44, consistent with typical MSC marker profiles. Other tested markers (CD14, CD26, CD31, CD34, CD45, CD73, CD79, HLA-DR) showed minimal or no expression (<5%).

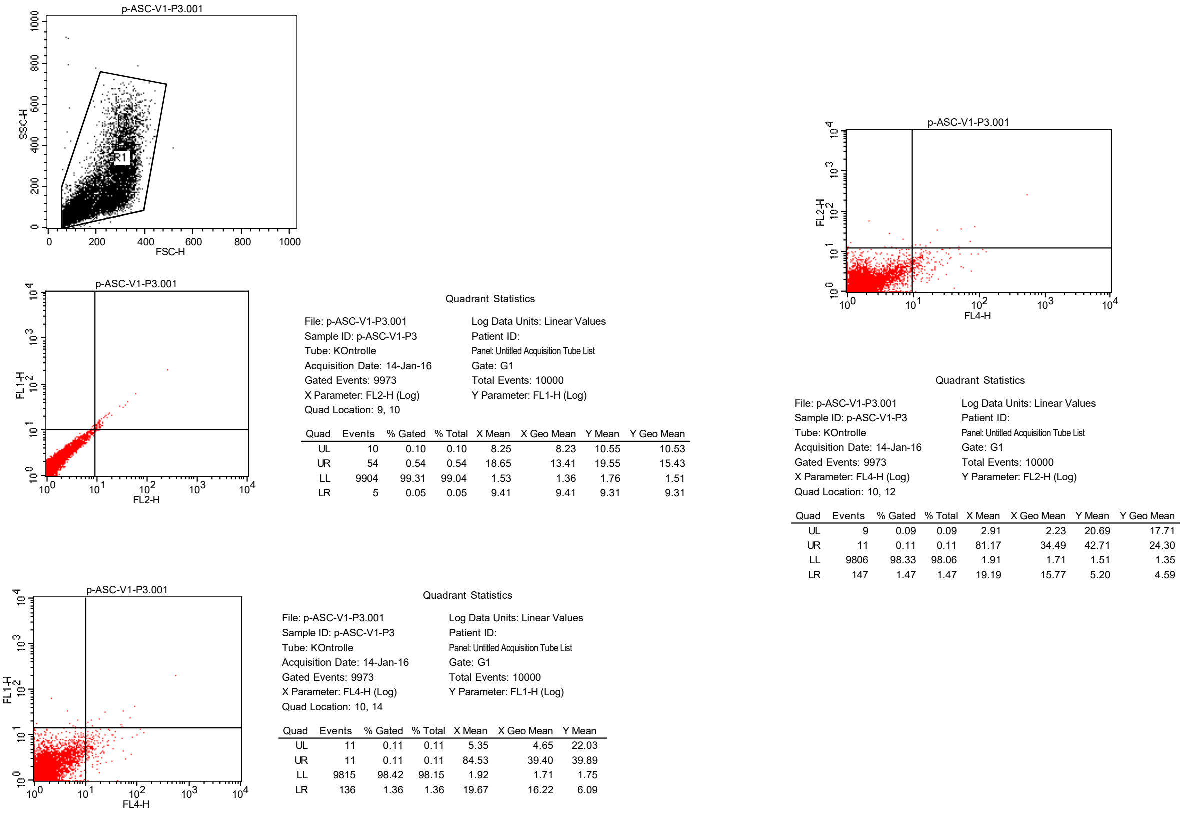

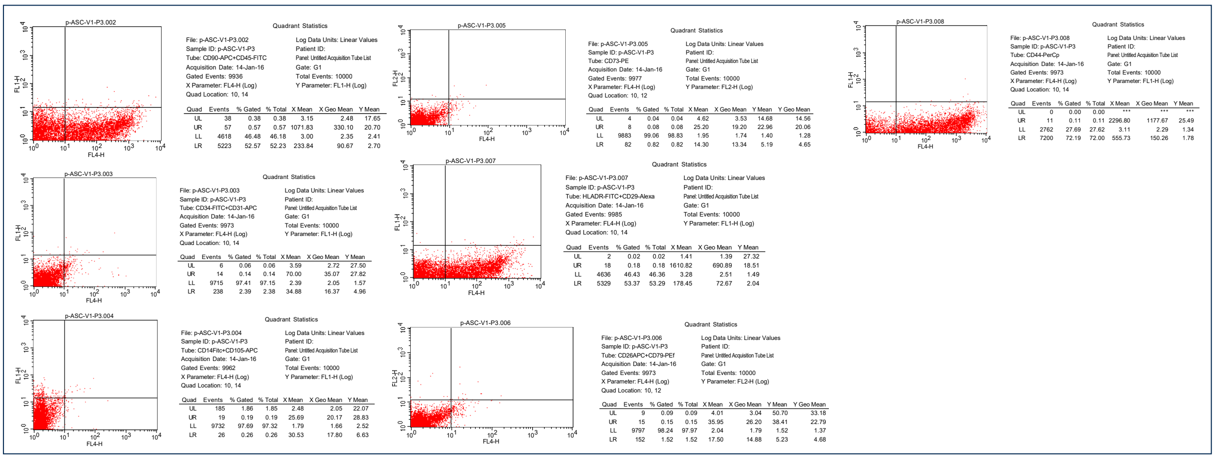

Figure S4: FACS- Gates of pASC

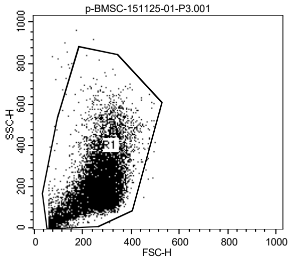

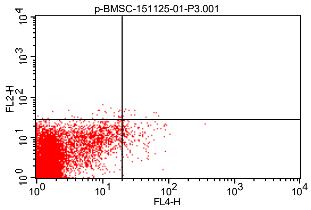

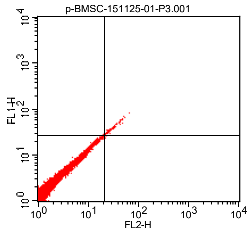

Quadrant Statistics

File: p-BMSC-151125-01-P3.001 | Log Data Units: Linear Values
Sample ID: p-BMSC-151125-01-P3 | Patient ID:
Tube: KOntrolle | Panel: Untitled Acquisition Tube List
Acquisition Date: 14-Jan-16 | Gate: G1
Gated Events: 9974 | Total Events: 10000
X Parameter: FL2-H (Log) | Y Parameter: FL1-H (Log)
Quad Location: 21, 26

Quad	Events	% Gated	% Total	X Mean	X Geo Mean	Y Mean	Y Geo Mean
UL	29	0.29	0.29	19.98	19.97	26.85	26.84
UR	136	1.36	1.36	28.58	27.68	36.23	35.15
LL	9805	98.31	98.05	4.04	2.84	5.16	3.49
LR	4	0.04	0.04	21.39	21.38	24.94	24.92

Quadrant Statistics

File: p-BMSC-151125-01-P3.001 | Log Data Units: Linear Values
Sample ID: p-BMSC-151125-01-P3 | Patient ID:
Tube: KOntrolle | Panel: Untitled Acquisition Tube List
Acquisition Date: 14-Jan-16 | Gate: G1
Gated Events: 9974 | Total Events: 10000
X Parameter: FL4-H (Log) | Y Parameter: FL2-H (Log)
Quad Location: 20, 28

Quad	Events	% Gated	% Total	X Mean	X Geo Mean	Y Mean	Y Geo Mean
UL	36	0.36	0.36	8.63	6.13	36.01	35.14
UR	13	0.13	0.13	35.31	31.67	38.98	38.44
LL	9770	97.95	97.70	2.34	1.81	4.14	2.85
LR	155	1.55	1.55	37.05	31.96	12.34	10.47

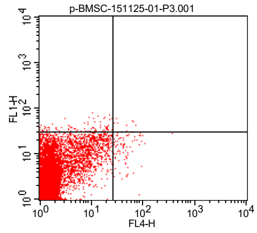

Quadrant Statistics

File: p-BMSC-151125-01-P3.001 | Log Data Units: Linear Valu
Sample ID: p-BMSC-151125-01-P3 | Patient ID:
Tube: KOntrolle | Panel: Untitled Acquisition Tube List
Acquisition Date: 14-Jan-16 | Gate: G1
Gated Events: 9974 | Total Events: 10000
X Parameter: FL4-H (Log) | Y Parameter: FL1-H (Log)
Quad Location: 26, 30

Quad	Events	% Gated	% Total	X Mean	X Geo Mean	Y Mean
UL	89	0.89	0.89	9.74	6.34	38.84
UR	11	0.11	0.11	48.95	46.59	41.65
LL	9795	98.21	97.95	2.45	1.84	5.25
LR	79	0.79	0.79	49.87	43.71	14.21

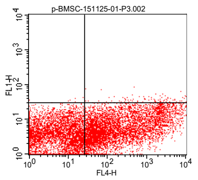

Quadrant Statistics

File: p-BMSC-151125-01-P3.002 | Log Data Units: Linear Valu
Sample ID: p-BMSC-151125-01-P3 | Patient ID:
Tube: CD90-APC+CD45-FITC | Panel: Untitled Acquisition Tube List
Acquisition Date: 14-Jan-16 | Gate: G1
Gated Events: 9983 | Total Events: 10000
X Parameter: FL4-H (Log) | Y Parameter: FL1-H (Log)
Quad Location: 26, 30

Quad	Events	% Gated	% Total	X Mean	X Geo Mean	Y Mean
UL	7	0.07	0.07	11.27	8.01	35.19
UR	96	0.96	0.96	2604.88	1238.85	37.29
LL	4626	46.34	46.26	7.86	4.69	4.83
LR	5254	52.63	52.54	713.83	210.07	7.54

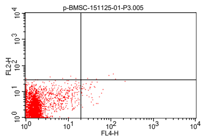

Quadrant Statistics

File: p-BMSC-151125-01-P3.005 | Log Data Units: Linear Values
Sample ID: p-BMSC-151125-01-P3 | Patient ID:
Tube: CD73-PE | Panel: Untitled Acquisition Tube List
Acquisition Date: 14-Jan-16 | Gate: G1
Gated Events: 3577 | Total Events: 3585
X Parameter: FL4-H (Log) | Y Parameter: FL2-H (Log)
Quad Location: 20, 28

Quad	Events	% Gated	% Total	X Mean	X Geo Mean	Y Mean	Y Geo Mean
UL	6	0.17	0.17	6.74	4.48	33.19	33.03
UR	5	0.14	0.14	81.72	70.67	35.90	35.23
LL	3530	98.69	98.47	2.10	1.71	3.44	2.41
LR	36	1.01	1.00	38.02	32.88	11.89	9.87

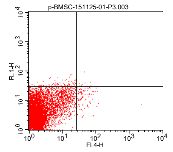

Quadrant Statistics

File: p-BMSC-151125-01-P3.003 | Log Data Units: Linear Valu
Sample ID: p-BMSC-151125-01-P3 | Patient ID:
Tube: CD34-FITC+CD31-APC | Panel: Untitled Acquisition Tube List
Acquisition Date: 14-Jan-16 | Gate: G1
Gated Events: 9982 | Total Events: 10000
X Parameter: FL4-H (Log) | Y Parameter: FL1-H (Log)
Quad Location: 26, 30

Quad	Events	% Gated	% Total	X Mean	X Geo Mean	Y Mean
UL	84	0.84	0.84	10.04	6.77	38.52
UR	9	0.09	0.09	45.53	43.71	41.71
LL	9793	98.11	97.93	2.56	1.92	4.77
LR	96	0.96	0.96	71.61	45.57	14.33

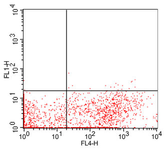

Quadrant Statistics

File: p-BMSC-Due8-P3.007 | Log Data Units: Linear Value
Sample ID: p-BMSC-Due8-P3 | Patient ID:
Tube: HLADR-FITC + CD29-Alexa | Panel: Untitled Acquisition Tube List
Acquisition Date: 25-Jan-16 | Gate: G1
Gated Events: 1883 | Total Events: 1890
X Parameter: FL4-H (Log) | Y Parameter: FL1-H (Log)
Quad Location: 19, 18

Quad	Events	% Gated	% Total	X Mean	X Geo Mean	Y Mean
UL	2	0.11	0.11	1.01	1.01	29.56
UR	17	0.90	0.90	842.01	415.06	29.42
LL	888	47.16	46.98	3.31	1.99	2.92
LR	976	51.83	51.64	634.29	261.19	4.11

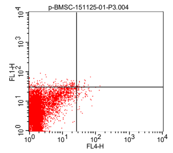

Quadrant Statistics

File: p-BMSC-151125-01-P3.004 | Log Data Units: Linear Valu
Sample ID: p-BMSC-151125-01-P3 | Patient ID:
Tube: CD14Fitc+CD105-APC | Panel: Untitled Acquisition Tube List
Acquisition Date: 14-Jan-16 | Gate: G1
Gated Events: 9990 | Total Events: 10000
X Parameter: FL4-H (Log) | Y Parameter: FL1-H (Log)
Quad Location: 26, 30

Quad	Events	% Gated	% Total	X Mean	X Geo Mean	Y Mean
UL	149	1.49	1.49	8.43	5.06	38.28
UR	23	0.23	0.23	44.49	40.02	37.52
LL	9762	97.72	97.62	2.35	1.81	6.59
LR	56	0.56	0.56	42.21	39.25	18.69

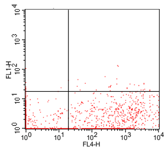

Quadrant Statistics

File: p-BMSC-Due8-P3.008 | Log Data Units: Linear Values
Sample ID: p-BMSC-Due8-P3 | Patient ID:
Tube: CD44-PerCp | Panel: Untitled Acquisition Tube List
Acquisition Date: 25-Jan-16 | Gate: G1
Gated Events: 1616 | Total Events: 1635
X Parameter: FL4-H (Log) | Y Parameter: FL1-H (Log)
Quad Location: 19, 18

Quad	Events	% Gated	% Total	X Mean	X Geo Mean	Y Mean
UL	10	0.62	0.61	3.84	1.71	34.03
UR	28	1.73	1.71	1528.88	672.52	35.22
LL	887	54.89	54.25	2.42	1.55	1.80
LR	691	42.76	42.26	1203.54	443.82	3.07

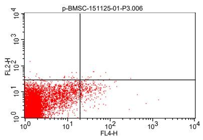

Quadrant Statistics

File: p-BMSC-151125-01-P3.006 | Log Data Units: Linear Values
Sample ID: p-BMSC-151125-01-P3 | Patient ID:
Tube: CD26APC+CD79-PEf | Panel: Untitled Acquisition Tube List
Acquisition Date: 14-Jan-16 | Gate: G1
Gated Events: 9983 | Total Events: 10000
X Parameter: FL4-H (Log) | Y Parameter: FL2-H (Log)
Quad Location: 20, 28

Quad	Events	% Gated	% Total	X Mean	X Geo Mean	Y Mean	Y Geo Mean
UL	28	0.28	0.28	7.06	4.55	40.22	37.67
UR	12	0.12	0.12	67.91	48.70	39.18	38.00
LL	9716	97.33	97.16	2.46	1.86	4.16	2.94
LR	227	2.27	2.27	49.05	35.43	11.96	10.33

Figure S5: FACS Gates of pBMSC

Flow cytometry was used to analyze the expression of characteristic MSC surface markers. pASCs and pBMSCs were stained with antibodies against CD29, CD90, CD44, CD14, CD26,

CD31, CD34, CD45, CD73, CD79, and HLA-DR, and analyzed using a dual-channel detection system (FL1-H and FL4-H).

Gating Strategy:
Initial gating was performed based on forward scatter (FSC) and side scatter (SSC) to exclude debris and cellular aggregates. Single cells were gated using FSC-A vs. FSC-H to ensure analysis of singlet populations. For marker evaluation, quadrant gating was applied to two-parameter dot plots (FL1-H vs. FL4-H). Quadrant thresholds were defined using fluorescence minus one (FMO) controls and unstained controls to accurately distinguish between negative and positive populations. The four quadrants represent:
LL ($FL1^-/FL4^-$): negative for both markers
LR ($FL1^+/FL4^-$): positive for marker detected in FL1
UL ($FL1^-/FL4^+$): positive for marker detected in FL4
UR ($FL1^+/FL4^+$): double-positive cells

Most cells were located in the LL quadrant, indicating a lack of marker expression. However, pASCs and pBMSCs showed high positivity for CD29, CD90, and CD44, with expression levels exceeding 70% in pASCs and 50% in pBMSCs, primarily observed in the LR or UR quadrants. In contrast, expression levels of CD14, CD26, CD31, CD34, CD45, CD73, CD79, and HLA-DR were below 5% in both cell types.

OD Readings on pASCs and pBMSCs

pASC + OM

	OM																													
0	0,069	0,088	0,194	0,086	0,152	0,102	0,203	0,213	0,196	0,196	0,222		0,201	0,228	0,204	0,25	0,248	0,812	0,078	0,077	0,059	0,075	0,074	0,092	0,038	0,043	0,041	0,043	0,046	0,045
7	0,305	0,371	0,17	0,147	0,109	0,18	0,175	0,155	0,132	0,118	0,136	0,162	0,208	0,303	0,359	0,339	0,289	0,29	0,099	0,101	0,057	0,111	0,075	0,076	0	0,021	0,014	0,016	0,009	0,013
14	0,128	0,143	0,34	0,366	0,289	0,507	0,209	0,195	0,192	0,193	0,231	0,234	0,429	0,421	0,443	0,393	0,36	0,424	0,038	0,043	0,047	0,05	0,067	0,035	0,079	0,058	0,075	0,07	0,073	0,085
21	0,188	0,609	0,142		0,303	0,285	0,254	0,21	0,265	0,221	0,229	0,229	0,587	0,473	0,608	0,496	0,523	0,565	0,073	0,053	0,075	0,082	0,072	0,058	0,283	0,095	0,094	0,097	0,112	0,118
28	0,397	0,527	0,229	0,248	0,257	0,291	0,219	0,249	0,228	0,264	0,235	0,296	0,596	0,091	0,178	0,208	0,479	0,419	0,133	0,089	0,113	0,093	0,117	0,197	0,113	0,248	0,247	0,197	0,156	0,133

pASC + OM + BMP-2

OM + BMP-2																			
0,069	0,088	0,194	0,152	0,078	0,077	0,059	0,075	0,074	0,092	0,203	0,213	0,196	0,196	0,222	0,201	0,228	0,25	0,248	0,812
0,393	0,34	0,139	0,149	0,221	0,188	0,204	0,224	0,22	0,245	0,129	0,214	0,22	0,212	0,215	0,441	0,474	0,485	0,507	0,381
1,21	0,756	0,197	0,423	0,155	0,185	0,2	0,202	0,244	0,174	0,52	0,787	0,623	1,085	0,648	0,593	0,488	0,578	0,568	0,618
2,37	4,262	0,983	2,292	2,549	0,37	0,323	2,355	0,408	3,421	6,07	6,43	3,77	1,24	2,17	7,41	3,98	5,35	1,98	1,42
6,68	8,415	5,261	2,716	4,984	5,972	5,616	6,876	4,012	5,456	12,55	13,84	12,99	7,09	6,51	9,38	10,3	4,5	8,32	10,09

pBMSC

	OM				OM+ BMP-2			
0	0,137	0,064	0,095	0,09	0,121	0,082	0,109	0,138
7	0,083	0,108	0,056	0,575	0,104	0,11	0,178	0,411
14	0,184	0,155	0,176	1,528	0,669	0,121	0,926	1,479
21	0,307	0,328	0,134	7,806	4,745	1,869	2,163	7,249
28	3,132	4,081	1,095	9,861	9,822	8,154	6,922	8,887

Table S1

This table presents estimated optical density (OD) values measured at 600 nm during Alizarin Red S staining to assess osteogenic differentiation in pASCs and pBMSCs cultured with or without BMP-2 supplementation. pASC cultures were measured 3 times to calculate a mean. Data points which were identified as negative or positive statistical outliers were excluded from the final analysis.

OD Readings on pASCs and pBMSCs under inhibitor supplementation

pASC

	Osteo + SB 431542						BMP-2 + SB431542					
1	0,074	0,124	0,143	0,161	0,16	0,127	0,09	0,164	0,163	0,165	0,195	0,157
3	0,054	0,16	0,15	0,15	0,156	0,116	0,053	0,16	0,173	0,206	0,151	0,115
7	0,251	0,3	0,096	0,136	0,075	0,103	0,338	0,273	0,128	0,138	0,103	0,102
14	0,072	0,065	0,279	0,44	0,232	0,135	0,151	0,18	0,269	0,445	0,241	0,267
21	0,121	0,536	0,147	0,198	0,191	0,131	0,451	1,689	2,335	3,79	2,47	0,235
28	0,44	0,389	0,268	0,179	0,241	0,138	2,34	7,065	2,411	3,023	2,217	2,879

	Osteo + Dorsomorphin						BMP-2 + Dorsomorphin					
1	0,093	0,115	0,143	0,157	0,181	0,119	0,104	0,141	0,182	0,17	0,196	0,136
3	0,049	0,128	0,17	0,178	0,145	0,151	0,048	0,2	0,191	0,203	0,102	0,118
7	0,281	0,304	0,084	0,108	0,085	0,093	0,317	0,325	0,125	0,137	0,11	0,111
14	0,076	0,094	0,231	0,473	0,166	0,251	0,164	0,204	0,208	0,366	0,346	0,295
21	0,132	0,502	0,15	0,069	0,212	0,201	0,528	2,292	0,273	0,302	0,343	0,309
28	0,291	0,428	0,176	0,228	0,188	0,141	1,424	2,426		0,231	0,377	0,427

	Osteo + BGJ398						BMP-2 + BGJ398					
1	0,12	0,115	0,186	0,154	0,215	0,117	0,123	0,136	0,148	0,122	0,204	0,186
3	0,069	0,168	0,206	0,186	0,238	0,19	0,08	0,277	0,201	0,207	0,167	0,105
7	0,328	0,361	0,092	0,151	0,074	0,104	0,44	0,362	0,131	0,118	0,14	0,057
14	0,123	0,122	0,331	0,227	0,36	0,445	0,4	0,324	0,273	0,188	0,23	0,209
21	0,286	0,431	0,125		0,262	0,446	0,957	0,732	2,775		1,935	0,113
28	0,412	0,508		0,24	0,221	0,32	3,447	3,337	3,007	2,484	7,47	1,744

pBMSC

	Osteo + SB 431542				BMP-2 + SB431542			
1	0,124	0,047	0,108	0,148	0,113	0,079	0,094	0,175
3	0,124	0,039	0,119	0,235	0,147	0,062	0,152	0,195
7	0,1	0,089	0,08	1,013	0,119	0,116	0,149	0,635
14	0,183	0,11	0,121	2,588	0,537	0,111	0,181	3,656
21	0,884	0,275	0,206	7,853	4,33	1,381	1,042	9,533
28	3,687	3,501	0,809	13,122	9,499	10,093	5,623	14,996

	Osteo + Dorsomorphin				BMP-2 + Dorsomorphin			
1	0,114	0,033	0,129	0,149	0,125	0,075	0,118	0,133
3	0,122	0,04	0,115	0,186	0,155	0,044	0,121	0,141
7	0,058	0,08	0,078	0,579	0,095	0,11	0,094	0,473
14	0,11	0,081	0,108	2,016	0,228	0,226	0,117	1,611
21	0,218	0,122	0,083	4,539	0,451	0,483	0,173	4,846
28	0,397	0,807	0,29	8,015	1,4	3,767	0,617	8,9

	Osteo + BGJ398				BMP-2 + BGJ398			
1	0,149	0,067	0,148	0,136	0,138	0,093	0,119	0,124
3	0,147	0,031	0,113	0,1	0,134	0,057	0,113	0,175
7	0,084	0,126	0,129	0,617	0,127	0,163	0,11	0,407
14	0,159	0,141	0,183	2,089	1,431	0,181	0,665	1,515
21	0,355	0,341	0,176	5,753	6,86	2,288	2,338	5,053
28	3,057	4,307	0,712	13,418	10,594	12,946	9,643	13,014

Table S2

This table presents estimated optical density (OD) values measured at 600 nm during Alizarin Red S staining to assess osteogenic differentiation in pASCs and pBMSCs cultured with or without BMP-2 supplementation and supplementation with one inhibitor (SB431542, Dorsomorphin, BGJ398)

OD Readings on pASCs and pBMSCs under supplementation of inhibitor combinations

pASC

	OM + SB431542 + Dorsomorphin			3MP-2 + SB431542 + Dorsomorphir		
14	0,266	0,274	0,81	0,372	0,752	0,27
21	0,638	0,487	0,866	2,762	1,496	0,57
28	0,836	0,611	1,438	4,841	1,28	0,825

	OM + SB431542 + BGJ398			BMP-2 + SB431542 + BGJ398		
14	0,202	0,548	0,995	1,26	1,112	0,413
21	0,667	0,827	1,063	1,51	4,132	0,628
28	0,802	0,782	1,64	1,883	4,828	1,663

	OM + Dorsomorphin + BGJ398			BMP-2 + Dorsomorphin + BGJ398		
14	0,171	0,47	0,805	0,348	0,647	0,434
21	0,733	0,735	1,125	1,893	1,614	1,08
28	0,699	0,896	1,674	2,609	1,681	1,934

pBMSC

	OM + SB431542 + Dorsomorphin			3MP-2 + SB431542 + Dorsomorphir		
14	1,896	2,688	1,201	4,946	5,388	2,432
21	2,044	3,96	4,688	6,068	9,864	4,992
28	3,026	3,852	6,222	10,804	7,436	11,148

	OM + SB431542 + BGJ398			BMP-2 + SB431542 + BGJ398		
14	2,833	1,611	4,098	7,418	8,008	3,862
21	4,656	2,41	5,402	8,26	17,624	10,416
28	6,666	3,094	5,594	10,688	20,144	14,448

	OM + Dorsomorphin + BGJ398			BMP-2 + Dorsomorphin + BGJ398		
14	1,901	2,155	1,614	3,474	2,005	2,386
21	3,526	4,676	2,652	9,288	4,86	9,844
28	3,268	5,424	3,038	10,444	11,556	5,888

Table S3

This table presents estimated optical density (OD) values measured at 600 nm during Alizarin Red S staining to assess osteogenic differentiation in pASCs and pBMSCs cultured with or without BMP-2 supplementation and supplementation with a combination of two inhibitors (SB431542, Dorsomorphin, BGJ398)

Western blot readings of key proteins expression during osteogenic differentiation of pASCs

smad4

	OM				OM+ BMP-2			
7-14	0,024314	0,012159	0,020206	0,029109	0,016573	0,017877	0,039794	0,018062
21-28	0,027913	0,023795	0,028924	0,015747	0,004476	0,005194	0,00821	0,000815

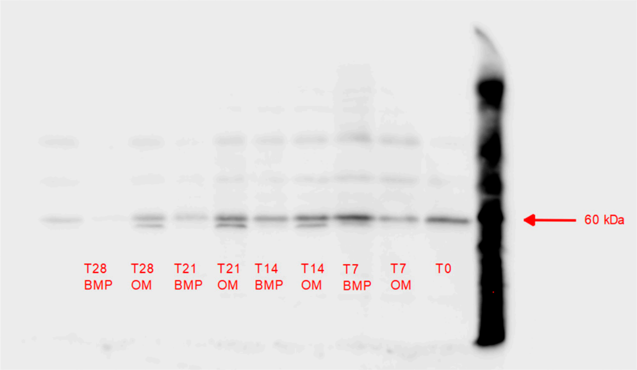

pp38

	OM				OM+ BMP-2			
7-14	0,004575	0,051467	0,046723	0,022679	0,127641	0,101979	0,078262	0,036971
21-28	0,082094	0,013147	0,01527	0,065327	0,055758	0,066931	0,01527	0,043196

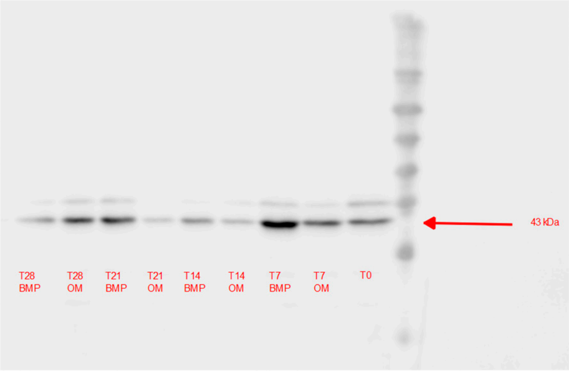

fgfr

	OM				OM+ BMP-2			
7-14	0,038616	0,176258	0,079431	0,095588	0,047004	0,053864	0,086773	0,193393
21-28	0,081478	0,160626	0,086327	0,094263	0,054261	0,027868	0,135975	0,084245

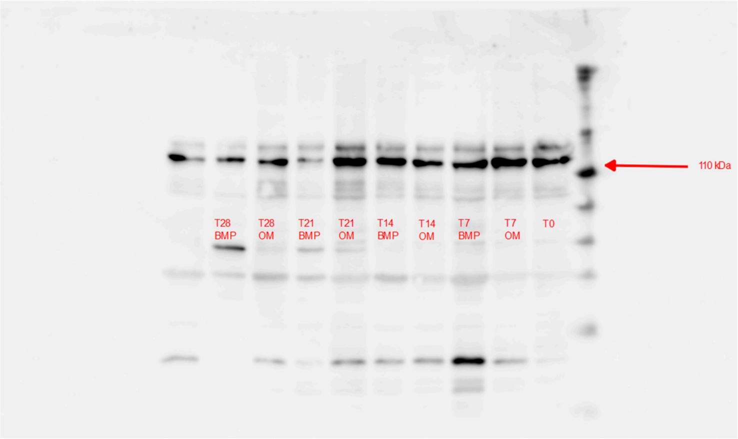

β-catenin

	OM				OM+ BMP-2			
7-14	0,018138	0,0255	0,005964	0,010857	0,393013	0,030421	0,017418	0,012889
21-28	0,104328	0,009358	0,10824	0,032253	0,159671	0,052947	0,267375	0,047698

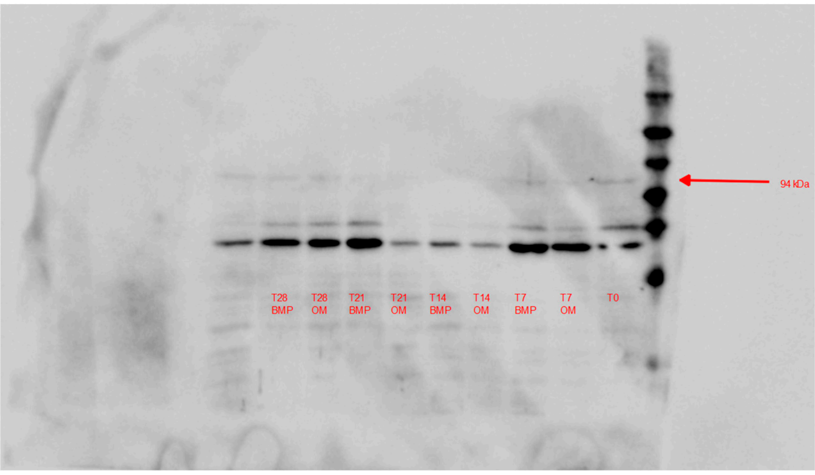

Readings of receptor expression during osteogenic differentiation of pASCs

ALK2

	OM												
0-7	37,61	27,09	22,25	6,93	16,93	32,78	24,87	12,84	19,56	15,94	9,66	60,46	9,18
9-17	37,38	6,99	43,27	43,21	53,22	15,23	21,98	32,16	31,33	10,8	9,08	15,23	13,1
19-28	22,74	6,07	8,04	5,28	16,99	1,44	33,18	22,05	11,26	5,72	7,21	35,25	4,7

	OM+ BMP-2														
0-7	2,28	21,92	38,79	1,68	15,25	32,71	27,26	9,78	24,09	27,64	12,69	37,61	27,09	22,25	3,14
9-17	23,94	62,59	46,57	25,04	52,27	57,94	24,5	23,63	41,47	14,07	21,22	26,55	7,18	44,39	48,95
19-28	10,34	41,76	3,04	54,24	7,67	41,39	59,5	4,27	11,57	39,12	6,92	56,23	3,91	20,46	83,43

ALK4

	OM													
0-7	86,64	82,5	97,88	67,69	94,56	97,69	83,87	93,54	87,15	94,95	58,94	67,44	90,9	93,77
9-17	75,2	86,68	57,91	99,23	6,19	64,68	34,67	4,8	70,95	6,01	4,24	64,71	3,07	3,69
19-28	38,8	25,32	3,16	9,45	6,54	89,26	31,64	2,27	4,19	5,03	2,91	3,82	11,53	3,32

	OM+ BMP-2															
0-7	86,64	82,5	97,88	1,78	79,64	57,78	97,84	63,79	88,09	85,53	22,79	98,17	29,75	70,88	36,93	66,3
9-17	89,94	67,4	65,11	61,46	99,63	9,27	92,78	47,19	6,23	90,79	61,72	2,07	9,91	92,37	1,73	4,65
19-28	54,49	14,76	4,33	85,28	3,33	5,3	79,54	19,72	4,22	3,73	3	2,85	8,12	5,25	5,85	2,6

ALK 6

	OM															
0-7	44,39	9,15	9,34	54,94	8,33	4,79	55,28	5,9	3,88	3,59	18,17	34,5	10,01	41,93	44,39	9,15
9-17	10,47	7,56	42,6	46,48	38,61	4,31	35,54	32,91	6,82	32,43	18,75	3,96	18,62	25,61	31,13	14,41
19-28	24,72	12,93	21,08	10,66	25,18	26,81	35,12	11,87	63,58	8,72	35,02	11,81	7,09	33,28	58,79	14,14

	OM+ BMP-2													
0-7	9,34	23,95	25,38	4,24	10,09	52,87	13,13	11,47	27,53	4,5	2,96	39,2	22,14	32,97
9-17	45,85	44,99	28,84	71,37	20,8	27,69	42,05	10,02	46,34	27,89	24,9	11,87	19,58	9,75
19-28	47,57	72,11	20,71	25,51	18,05	59,44	14,81	63,53	61,76	18,72	50,03	14,49	38,5	49,8

ALK5

	OM													
0-7	78,19	20,4	23,93	70,88	14,76	28,23	73,87	9,86	9,54	11,19	24,59	51,46	10,17	42,99
9-17	49,29	3,62	40,81	47,75	36,18	9,67	39,98	34,89	13,89	35,87	24,26	8,64	19,73	37,56
19-28	31,66	17,77	26,87	16,74	30,45	23,23	37,4	13,31	65,06	9,73	52,27	17,3	9,83	40,22

	OM+ BMP-2															
0-7	78,19	20,4	23,93	36,54	37,11	15,42	33,1	67,42	15,95	7,92	30,13	7,04	4,85	42,71	21,26	31,38
9-17	41,91	16,17	52,54	52,06	32,27	74,06	52,05	26,46	58,9	19,79	57,36	28,36	43,24	33,25	18,77	12,07
19-28	64,68	21,77	63,02	73,28	34,47	58,07	19,81	74,7	31,08	78,24	76,01	32,38	54,01	35,56	38,56	55,78

ALK7

	OM													
0-7	85,89	24,91	23,1	75,25	22,79	31,93	72,57	17,18	33,94	10,4	20,51	65,07	12	58,44
9-17	35,73	7,36	48,15	55,37	53,4	10,02	46,55	44,16	9,42	41,19	24,48	9,43	25,18	41,39
19-28	42,47	26,36	1,54	18,74	38,13	33,44	59,63	2,35	65,97	16,35	59,88	19,55	3,51	42,32

	OM+ BMP-2															
0-7	85,89	24,91	23,1	42,31	56,32	17,43	33,94	74,37	17,97	21,41	30,95	4,91	4,91	23,19	37,31	46,85
9-17	36,12	14,6	64,4	36,29	37,54	80,69	38,23	45,42	61,14	12,32	69,94	36,91	44,55	28,59	30,44	47,82
19-28	67,52	24,67	1,62	71,27	38,6	46,24	26,81	74,54	1,78	69,64	80,81	35,86	60,39	28,95	48,32	58,3

BMPRII

	OM											
0-7	1,43	14,57	45,51	17,51	5,23	16,23	5,03	8,77	24,59	6,34	16,31	2,24
9-17	9,53	4,11	34,06	33,54	58,21	17,93	18,51	20,2	20,26	6,93	6,01	11,01
19-28	14,24	4,64	9,97	5,09	4	1,16	31,38	8,54	0,58	7,94	2,57	0,27

	OM+ BMP-2											
0-7	1,43	14,57	45,51	8,96	15,24	20,66	11,64	9,94	6,77	12,84	23,2	11,38
9-17	5,81	1,36	28,91	77,31	31,13	18,52	14,73	9,06	7,31	9,61	0,22	6,61
19-28	5,64	13,45	46,58	21	3,1	6,4	33,09	36,68	11,35	3,7	30,78	11,62

Donor Overview

Origin	Number of Donors	Harvested Tissue	Isolated Cells	Species and Breed	Age
Viersen	15	Skin with subcutaneous adipose tissue	pASCs	Domestic pig (Danish Landrace sow, Pietrain boar)	6 months +/- 2 weeks
Düsseldorf	6	Skin with subcutaneous adipose tissue, bone marrow	pASCs, pBMSCs	Göttingen Minipig	1.5 years

Table S4 - Donor Overview

Experimental Design and Replicate Overview

**Experiment / Assay**	**Cell Type(s)**	**Number of Biological Replicates (Donors)**	**Number of Technical Replicates (per donor)**	**Independent Experiments**	**Notes**
BMP-2 Supplementation Study: Alizarin Red S Quantification (OD600)	pASC, pBMSC	pASC: 18 (15 Viersen, 3 Düsseldorf) pBMSC: 6 (Düsseldorf)	3 wells per donor	2	OM vs. OM + BMP-2 conditions/ OD measured at days 0, 7, 14, 21, 28
Inhibitor studies: Alizarin Red S Quantification (OD600)	pASC, pBMSC	pASC: 6 donors pBMSC: 4 donors	3 wells per donor	12	OM (+ SB431542, Dorsomorphin, BGJ398) vs. OM + BMP-2 (+

					SB431542, Dorsomorphin, BGJ398)
Co- Inhibition studies: Alizarin Red S Quantification (OD600)	pASC, pBMSC	pASC: 6 donors pBMSC: 4 donors	3 wells per donor	12	OM vs. OM + BMP-2 (suppl. SB431542+ Dorsomorphin/ SB431542 + BGJ398/ Dorsomorphin+ BGJ398)
Flow Cytometry (CD Antigen Panel)	pASC, pBMSC	pASC: 12 pBMSC: 4	1–2 measurements per donor	1	For MSC marker validation
Western Blot (Smad4, pp38, ß-catenin, fgfr)	pASC	6 donors	Cell culture samples were analyzed randomly	4	GAPDH as reference; measured at days 7, 14, 21, 28
Flow Cytometry (ALK2,4,6,5,7 and BMPRII Antigen Panel)	pASC	6 donors	Cell culture samples were analyzed randomly	6	for evaluation of receptor epression in the course of osteogenic differentiation
Microscopy	pASC, pBMSC	4–6 donors	representative images shown (Figure 1&3)	1	Staining at 7, 14, 21, 28 days of differentiation

Table S5: Experimental Design and Replicate Overview
